# The lncRNA RUNX1-IT1 regulates C-FOS transcription by interacting with RUNX1 in the process of pancreatic cancer proliferation, migration and invasion

**DOI:** 10.1038/s41419-020-2617-7

**Published:** 2020-06-02

**Authors:** Songsong Liu, Junfeng Zhang, Liangyu Yin, Xianxing Wang, Yao Zheng, Yujun Zhang, Jianyou Gu, Ludi Yang, Jiali Yang, Ping Zheng, Yan Jiang, Ling Shuai, Xiongwei Cai, Huaizhi Wang

**Affiliations:** 1 Institute of Hepatopancreatobiliary Surgery, Southwest Hospital, Third Military Medical University (Army Medical University), Chongqing, P. R. China; 2 Institute of Hepatopancreatobiliary Surgery, Chongqing General Hospital, University of Chinese Academy of Sciences, Chongqing, P. R. China; 3 Department of First Hepatobiliary Surgery, Zhujiang Hospital, Southern Medical University, Guangzhou, Guangdong P. R. China; 4 Department of Obstetrics and Gynecology, Southwest Hospital, Third Military Medical University (Army Medical University), Chongqing, P. R. China

**Keywords:** Cancer epidemiology, Medical research

## Abstract

Numerous long noncoding RNAs (lncRNAs) are aberrantly expressed in pancreatic cancer (PC); however, their functions and mechanisms in cancer progression are largely unknown. In this study, we identified a novel PC-associated lncRNA, RUNX1-IT1, that was significantly upregulated in PC patient samples from multiple centers and associated with poor prognosis. In vitro and in vivo, alterations in RUNX1-IT1 expression markedly affected PC proliferation, migration and invasion. RUNX1-IT1 contributed to the progression of PC by interacting with the adjacent gene RUNX1. Rescue experiments showed that RUNX1 reduced the cancer-promoting effect of RUNX1-IT1. RNA-seq analysis after silencing RUNX1-IT1 and RUNX1 highlighted alterations in the common target C-FOS. Mechanistically, we demonstrated that RUNX1-IT1 was a trans-acting factor that participated in the proliferation, migration and invasion of PC by recruiting RUNX1 to the C-FOS gene promoter. Furthermore, RUNX1-IT1 enhanced the transcription of the RUNX1 gene, indicating its potential as a cis-regulatory RNA involved in the upstream regulation of RUNX1. Overall, RUNX1-IT1 is a crucial oncogenic lncRNA that activates C-FOS expression by regulating and recruiting RUNX1 and is a potential prognostic biomarker and therapeutic target for PC.

## Introduction

The efficacy of clinical pancreatic cancer (PC) treatments is extremely poor, and the 5-year survival rate is only approximately 7%^1^. PC has become one of the most difficult malignant tumors to treat, with the worst prognosis, and its mortality rate is predicted to become the second highest by 2030^2,3^. The poor overall prognosis of patients with PC is mainly due to early distant metastasis, late diagnosis, and ineffective clinical treatment^4–7^. Therefore, improving our understanding of the molecular mechanisms underlying the progression of PC and identifying novel therapeutic targets are necessary.

In recent years, long noncoding RNAs (lncRNAs) have been found to play an important role in the development of tumors^8,9^. Recent studies have also demonstrated that many lncRNAs are dysregulated in PC, and several of these have been well characterized^10,11^. For example, ENST00000480739 expression is downregulated in PC, which is negatively correlated with the TNM stage and lymph node metastasis. ENST00000480739 suppresses tumor cell invasion by regulating OS-9 in PC^12^. GLS-AS is downregulated in PC cells, and depletion of GLS-AS promotes PC cell proliferation and invasion both in vitro and in vivo. GLS-AS, a potential therapeutic target for metabolic reprogramming in PC, is a critical regulator of a feedback loop between Myc and GLS^13^. AGAP2-AS1 epigenetically inhibits the expression of ANKRD1 and ANGPTL4 by recruiting zeste homolog 2, thereby promoting PC proliferation and metastasis^14^. LINC00673 levels are significantly lower in PC cells and tissues than in normal cells and tissues. A G > A mutation at rs11655237 in exon 4 of LINC00673 creates a target miR-1231 binding site, which diminishes the effect of LINC00673 in an allele-specific manner and thus confers susceptibility to tumorigenesis^15^. In summary, lncRNAs are involved in the development of PC through various mechanisms.

However, many lncRNAs are still being discovered and have yet to be annotated. Using microarrays, we herein first explored and identified the differentially expressed lncRNAs between PC and normal pancreatic (NP) tissues. We found that the lncRNA RUNX1-IT1 was significantly high expressed in cancerous tissues and closely associated with PC progression. However, the research of RUNX1-IT1 in tumors is relatively lacking, and its role and mechanism in PC have not been reported. RUNX1-IT1 is transcribed from the intron of the RUNX1 gene, which is an important transcription factor in the hematopoietic system^16^. RUNX1 plays an important role in solid tumors, and its abnormal expression is closely associated with cancer progression^17,18^. Few reports have addressed the role of RUNX1 in PC, and the latest study demonstrated that RUNX1 could promote the invasion and metastasis of PC cells by regulating miR-93^19^. However, due to the lack of systematic research, the deeply regulatory mechanism of RUNX1 in PC are unclear. Here, we found that the expression of RUNX1-IT1 and RUNX1 was highly correlated and we thus questioned whether they have similar biological functions in PC. Therefore, this study focused on whether RUNX1-IT1 has an important biological effect in PC by collaborating with the transcription factor RUNX1.

## Materials and methods

### Tissue samples

Eighty-three freshly frozen PC samples (38 with adjacent noncancerous tissues) and 16 NP tissue samples were obtained from the Institute of Hepatopancreatobiliary Surgery, Southwest Hospital, Army Medical University (the NP samples were obtained from organ donors). The tissue microarrays comprised 175 formalin-fixed paraffin-embedded tissue samples that were acquired from the archived collections of Southwest Hospital, Army Medical University (91 PC and 13 NP samples), Wuhan Tongji Hospital (34 PC samples) and Soochow University (50 PC samples). None of the patients had received radiotherapy or chemotherapy before surgery. The study was approved by the Ethics Committee of Southwest Hospital of Chongqing.

### Cell lines

The human PC cell lines AsPC-1, BxPC-3, CFPAC-1, PANC-1 and SW1990 (ATCC, Manassas, VA, USA) were cultured in complete growth medium with 10% fetal bovine serum (Gibco, USA), as recommended by the manufacturer. Human pancreatic epithelial ductal cells were purchased from GENNIO BIO (Guangzhou, China). Cultured cells were maintained at 37 °C in a humidified incubator with 5% CO_2_. All cell lines were fingerprinted for authenticity validation.

### Statistical analysis

Continuous data are presented as the mean ± standard deviation (SD) and were compared using a *t* test. Categorical data are expressed as numbers (percentages) and were compared using the chi-squared test. Continuous data with three or more groups were compared using one-way ANOVA, followed by Tukey’s post hoc analysis. The normality of continuous data was evaluated using the Shapiro-Wilk test. Correlations between two variables were analyzed by the Pearson’s or Spearman’s correlation method as appropriate. The Kaplan–Meier method was used to analyze overall survival. Cox regression was used for multivariate survival analysis. All statistical analyses were performed using SPSS 17.0 statistical software (SPSS Inc., Chicago, IL, USA).

### Supplemental materials and methods

The Supplemental Materials and Methods are provided in Additional file 1: Supplementary Table S1 and Additional file 2: Supplementary Table S2.

## Results

### RUNX1-IT1 expression is significantly upregulated in PC

**Fig. 1:**
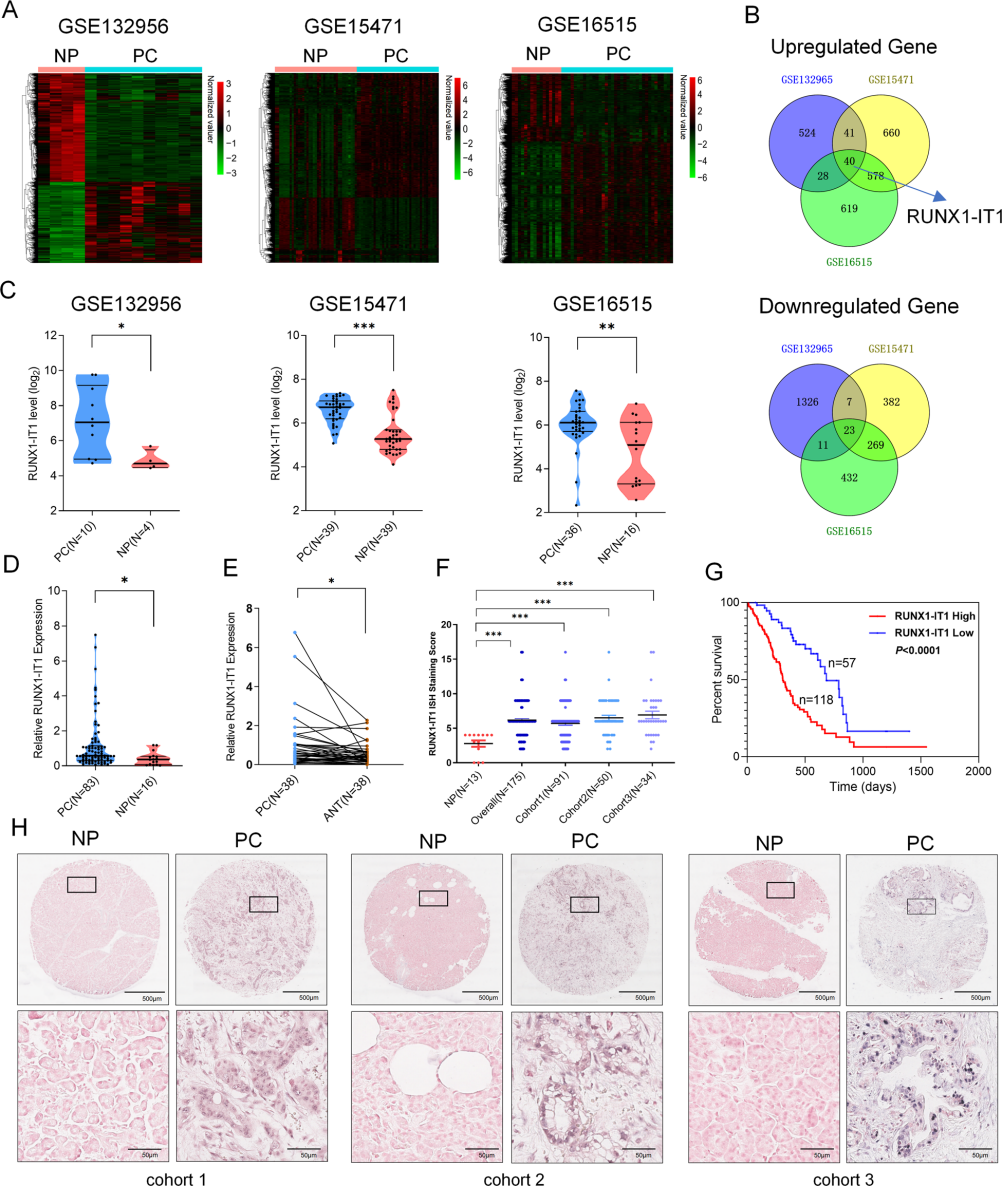
LncRNA RUNX1-IT1 is overexpressed in PC. **a** Hierarchical clustering analysis of genes that are differentially expressed in pancreatic cancer (PC) and normal pancreatic (NP) tissues (fold change: top 2000; *P* < 0.05). **b** Venn diagram of upregulated and downregulated genes in the three GEO datasets. **c** Expression profile of RUNX1-IT1 in the three GEO datasets. **d** RUNX1-IT1 expression was assessed by qRT-PCR in PC and NP tissues. **e** RUNX1-IT1 expression was assessed by qRT-PCR in paired PC tissues. **f** The RUNX1-IT1 expression in microarrays comprising PC (*N* = 175) and NP (*N* = 13) samples from three independent cohorts was assessed by ISH. **g** Kaplan–Meier analysis of the overall survival of PC patients based on the RUNX1-IT1 ISH score data. **h** Representative images of RUNX1-IT1-positive cells in PC and NP tissue samples from the three independent cohorts (scale bars, 500 and 50 μm) (*P < 0.05, **P < 0.01, ***P < 0.001).

To investigate lncRNA expression in PC, we first performed a gene microarray (Additional file 3: Supplementary Fig. S1) analysis comparing PC and NP tissues, and the data were uploaded to Gene Expression Omnibus (GEO: GSE132956). Microarray profiles comprising data from two other research centers were analyzed (GSE16515 and GSE15471, Fig. 1a and Additional file 4: Supplementary Table S3). Through an integrative analysis of microarrays, we found 40 upregulated genes and 23 downregulated genes in PC tissues compared with NP tissues (Fig. 1b). We then focused on the 40 upregulated genes because of their enhanced potential for use as early diagnostic markers or intervention targets. Interestingly, RUNX1-IT1 was the only lncRNA among the 40 upregulated genes, as the others were encoded proteins (Additional file 5: Supplementary Table S4); thus, its expression profile in the microarrays was analyzed (Fig. 1c). According to NCBI (gene NR_026812.1), the RUNX1-IT1 has only one transcript, and we chose to focus on this lncRNA given that it neighbors RUNX1 and that its function and mechanism in PC have not been reported. The online coding potential assessment tool analysis of lncRNA coding predictions showed that RUNX1-IT1 lacks protein-coding capacity (coding probability: 0.17; CP < 0.364 indicates noncoding sequences; http://lilab.research.bcm.edu/cpat/).

To further corroborate these findings, we assessed RUNX1-IT1 expression in PC, NP and paired adjacent nontumor (ANT) tissues by qRT-PCR. RUNX1-IT1 was markedly upregulated in PC tissues compared with ANT and NP tissues, consistent with our prior observation of RUNX1-IT1 expression in microarrays (Fig. 1d, e).

Next, we analyzed tissue microarrays comprised of 175 PC tissues and 13 NP tissues from 3 centers using in situ hybridization (ISH). The ISH staining score standard and representative positively stained images are shown in Additional file 6: Supplementary Fig. S2. RUNX1-IT1 expression was assessed in three independent PC cohorts, and statistical analyses revealed that RUNX1-IT1 was markedly upregulated in three independent PC cohorts and overall PC samples but expressed at low levels in NP tissues (Fig. 1f, h).

### RUNX1-IT1 expression is correlated with PC progression and poor prognosis

To investigate the correlation between RUNX1-IT1 expression and the clinical characteristics of PC, we divided the 175 samples into two groups according to the ISH staining score. Statistical analyses revealed that RUNX1-IT1 expression was positively correlated with the tumor differentiation grade (*P* = 0.005), lymph node invasion (*P* = 0.001) and clinical stage (*P* = 0.007) in PC patients (Additional file 7: Supplementary Table S5). Survival analysis was performed to assess the 175 PC patients after surgical resection, and high RUNX1-IT1 expression was closely correlated with a dramatic decrease in overall survival (Fig. 1g). In addition, univariate analysis showed that in patients, poor differentiation, lymph node invasion, advanced clinical stage and increased RUNX1-IT1 expression were significantly associated with an increased risk of cancer-related death. Multivariate Cox regression analyses showed that tumor differentiation (*P* < 0.05), clinical stage (*P* = 0.002) and RUNX1-IT1 expression (*P* < 0.001) were independent prognostic factors (Additional file 8: Supplementary Table S6). These data suggest that RUNX1-IT1 expression may play an important role in PC progression.

### Knockdown of RUNX1-IT1 significantly inhibits PC cell proliferation, migration and metastasis in vitro and in vivo

**Fig. 2:**
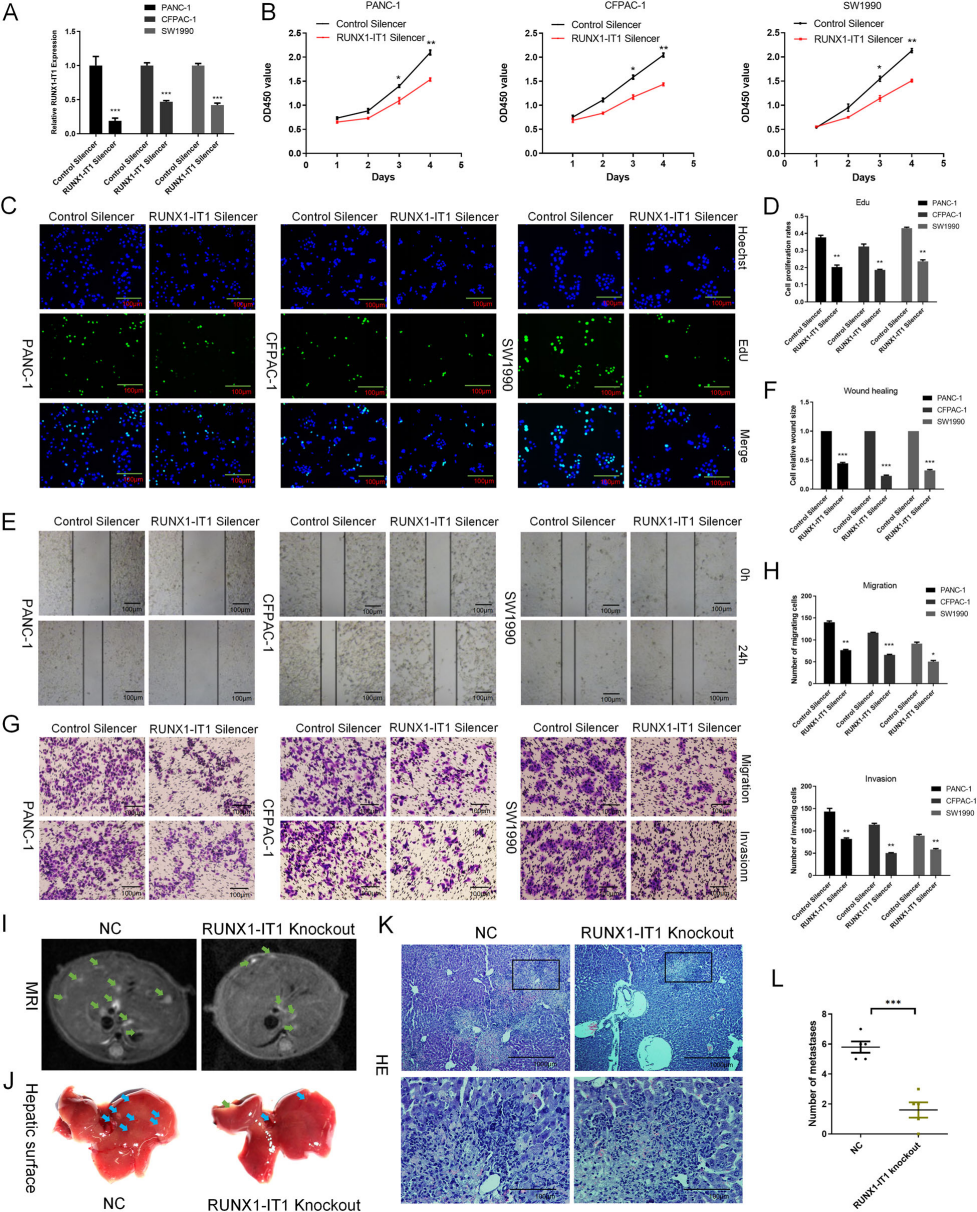
Knockdown of RUNX1-IT1 significantly inhibits PC cell proliferation, migration and metastasis in vitro and in vivo. **a** qRT-PCR analysis showed that RUNX1-IT1 expression was knocked down in PANC-1, CFPAC-1 and SW1990 cells by Smart Silencer lncRNA (RUNX1 Silencer and Control Silencer). **b** CCK8 assays were used to assess the viability of two groups of PC cells transfected with either RUNX1-IT1 Silencer or Control Silencer. **c**, **d** EdU assays were used to assess the cell proliferation ability. **e**, **f** Migration ability was assessed by a wound healing assay. **g**, **h** Transwell assays were used to assess the cell migration and invasion abilities. **i** Abdominal MRI scans of nude mice after 6 weeks of orthotopic PC xenograft growth. The livers of nude mice in the RUNX1-IT1 knockout PANC-1 cell group showed few metastatic lesions, but obvious metastatic lesions were found in the livers of the control group mice (CRISPR-Cas9 lentiviral system). **j** Images of micrometastases on the liver surface in the RUNX1-IT1 knockout PANC-1 group and in the control group. **k** HE staining of liver metastatic lesions was performed. The representative HE images are shown (scale bars, 1000 and 100 μm). **l** Histogram indicating the numbers of metastasized lesions in the two groups of mice. The number of metastatic lesions in the RUNX1-IT1 knockout PANC-1 group (*N* = 5) was significantly decreased compared with that in the control group (*N* = 5) (*P < 0.05, **P < 0.01, ***P < 0.001).

To investigate the biological function of RUNX1-IT1 in PC, we performed loss-of-function studies in PC cells. First, RUNX1-IT1 was found to be higher in PC cells than in pancreatic ductal epithelial cell (Additional file 9: Supplementary Fig. S3a). Then, RUNX1-IT1 5′ and 3′ RACE were performed to investigate full-length RUNX1-IT1 in PC cells (Additional file 9: Supplementary Fig. S3b, c). We performed loss-of-function experiments using Smart Silencer lncRNA (Ribo, China), which comprises a mixture of antisense oligonucleotides and siRNAs, and successful knockdown of RUNX1-IT1 was confirmed by qRT-PCR (Fig. 2a). Cell proliferation experiments (CCK8 and EdU) showed that RUNX1-IT1 knockdown suppressed PC cell proliferation (Fig. 2b–d). The migration and invasion abilities of transfected PC cells were assessed by wound healing and transwell assays. In the wound healing assay, knockdown of RUNX1-IT1 dramatically inhibited cell migration (Fig. 2e, f). Accordingly, in the transwell assay, PC cell migration and invasion were significantly decreased by RUNX1-IT1 knockdown (Fig. 2g, h). To further validate the function of RUNX1-IT1 in vivo, we used the CRISPR-Cas9 lentiviral system to successfully construct RUNX1-IT1 knockout (via sgRNA) and control PANC-1 cells (Additional file 9: Supplementary Fig. S3d). A total of 10^6^ stably transfected PANC-1 cells were injected into the distal pancreatic tissues of 4- to 6-week-old nude mice to assess whether RUNX1-IT1 influences PC cell progression in vivo. After 6 weeks, a small-animal MRI instrument was used to scan the mouse abdomens. A few cases of intrahepatic metastasis were found in each of the 5 mice in the RUNX1-IT1 knockout PANC-1 cell group, while extensive intrahepatic metastasis was observed in the control PANC-1 cell group (Fig. 2i). The liver surfaces of mice in the RUNX1-IT1 knockout PANC-1 cell group exhibited fewer micrometastatic lesions than those in the control group (Fig. 2j, l). Moreover, hematoxylin and eosin (HE) staining showed liver metastatic lesions in the RUNX1-IT1 knockout PANC-1 cell group and the control group (Fig. 2k).

### LncRNA RUNX1-IT1 is associated with the transcription factor RUNX1

**Fig. 3:**
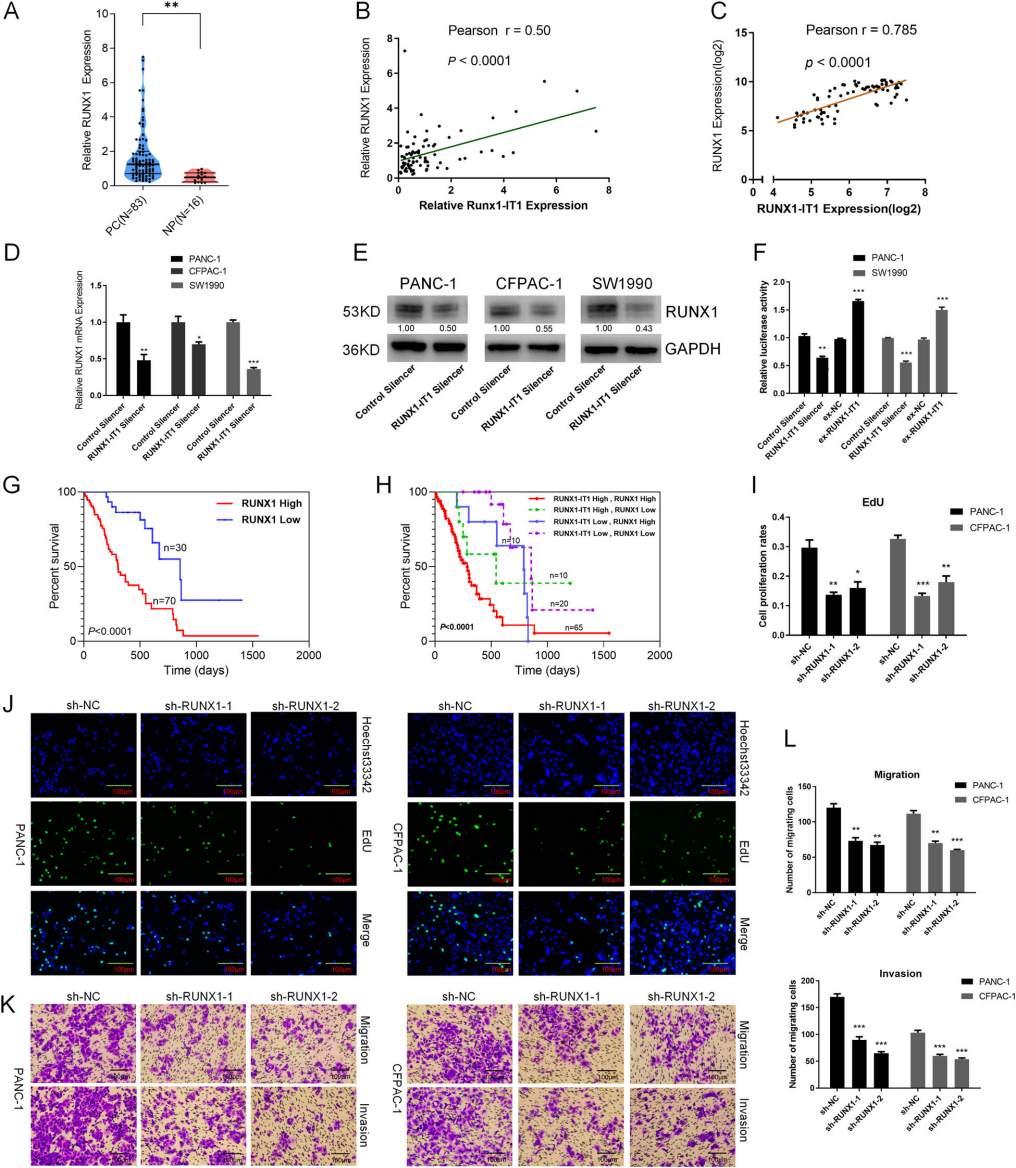
LncRNA RUNX1-IT1 expression is associated with the transcription factor RUNX1. **a** RUNX1 expression was assessed by qRT-PCR in PC and NP tissues. **b** Correlation analysis of RUNX1-IT1 and RUNX1 expression in PC tissues by qPCR. **c** Correlation analysis of RUNX1-IT1 and RUNX1 using GEO data (GSE15471). **d** RUNX1 mRNA expression levels in RUNX1-IT1 knockdown and control cells were analyzed by qPCR. **e** The RUNX1 protein expression levels in RUNX1-IT1 knockdown and control cells were measured by WB. **f** Luciferase activity assays were performed on RUNX1-IT1 knockdown and RUNX1-IT1-overexpressing PANC-1 and SW1990 cells cotransfected with the pGL3 reporter vector containing the WT RUNX1 promoter (RUNX1 promoter length: 2200 bp from the transcription start site (TSS)). **g** Kaplan–Meier survival analysis of PC patients based on RUNX1 IHC score data. **h** Kaplan–Meier survival analysis of the four groups of PC patients based on RUNX1-IT1 ISH and RUNX1 IHC datas. **i**, **j** EdU assays were used to assess the proliferation ability of PANC-1 and SW1990 cells transfected with sh-RUNX1-1, sh-RUNX1-2, and control lentiviral vectors. **k**, **l** Transwell assays were used to assess the migration and invasion abilities of PANC-1 and SW1990 cells transfected with sh-RUNX1-1, sh-RUNX1-2 and control lentiviral vectors (*P < 0.05, **P < 0.01, ***P < 0.001).

Interestingly, RUNX1-IT1 is transcribed from the intron of the RUNX1 gene, which is an important transcription factor involved in the development of tumors. We first performed qPCR to detect RUNX1 expression in PC and NP tissues, revealing markedly upregulated mRNA expression in PC compared with NP tissues (Fig. 3a). Correlation analysis showed that RUNX1-IT1 and the adjacent gene RUNX1 were positively coexpressed in PC patients (Fig. 3b), and this was validated by GEO data (Fig. 3c). Then, qPCR and western blot (WB) assays were performed to investigate whether RUNX1-IT1 influenced RUNX1 expression. Knockdown of RUNX1-IT1 reduced the mRNA and protein expression levels of RUNX1 (Fig. 3d, e). Furthermore, we performed dual luciferase reporter gene assays to investigate whether RUNX-IT1 regulated RUNX1 expression by affecting the transcriptional activity of the RUNX1 promoter. We selected the RUNX1 proximal promoter because it drives the most highly expressed form, RUNX1b^20^. Compared with the control, the group cotransfected with the RUNX1 promoter vector and RUNX1-IT1 Silencer exhibited significantly decreased luciferase activity, and the opposite pattern was observed in the RUNX1-IT1 overexpression group (Fig. 3f). This result indicated that RUNX1-IT1 may regulate RUNX1 transcription. Next, we performed RUNX1 immunohistochemistry (IHC) assays and statistical analysis indicated high RUNX1 expression was closely correlated with a dramatic decrease in overall survival (Fig. 3g). Importantly, Kaplan–Meier survival analysis of subgroups showed that the group with high expression levels of RUNX1-IT1 and RUNX1 had the worst prognosis (Fig. 3h). To further investigate whether RUNX1 has similar biological functions in PC, we constructed stable RUNX1 knockdown PANC-1 and SW1990 cells via a lentiviral infection system (Additional file 9: Supplementary Fig. S3e, f), and EdU and transwell assays indicated that knockdown of RUNX1 inhibited PC cell proliferation, migration and invasion (Fig. 3i–l).

A previous study demonstrated that lncRNAs epigenetically activate gene expression by modifying H3K27 acetylation in PC^12^. We searched the UCSC genome database (http://genome.ucsc.edu/) and found that the RUNX1 promoter region may be modified by H3K27 acetylation (Additional file 10: Supplementary Fig. S4a). We further explored whether the lncRNA RUNX1-IT1 upregulated RUNX1 expression by modifying the H3K27 acetylation of the RUNX1 gene proximal promoter. ChIP-PCR analysis showed that H3K27 acetylation modification may have occurred in the RUNX1 promoter region (Additional file 10: Supplementary Fig. S4b), and knockdown of lncRNA RUNX1-IT1 significantly decreased the level of H3K27 acetylation of the RUNX1 proximal promoter (Additional file 10: Supplementary Fig. S4c). These results indicate that the lncRNA RUNX1-IT1 may have a cis-acting regulatory effect on the RUNX1 proximal promoter.

### RUNX1-IT1 promotes PC cell progression through RUNX1 in vitro and in vivo

**Fig. 4:**
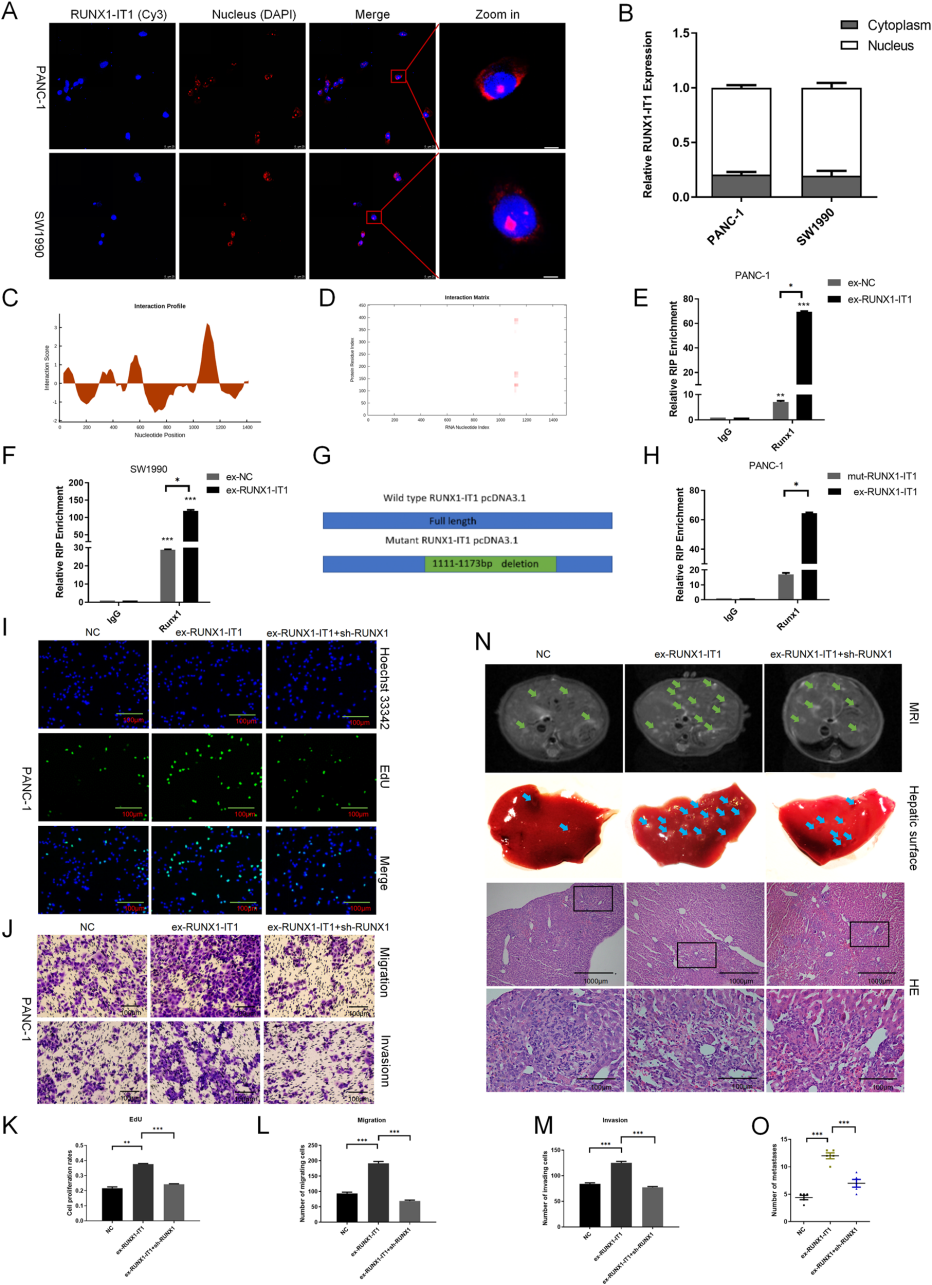
RUNX1-IT1 functions via the transcription factor RUNX1 in PC. **a** FISH analysis showing the subcellular localization of RUNX1-IT1 in PANC-1 and SW1990 cells. **b** Histogram showing the expression level of RUNX1-IT1 in the subcellular fractions of PANC-1 and SW1990 cells, as analyzed by qPCR. **c** The interaction profile, which represents the protein interaction score (*Y* axis) relative to the RUNX1-IT1 RNA sequence (*X* axis), provides information about the region most likely to be bound by the protein. **d** The interaction matrix, which shows a heatmap of the RUNX1 protein (*Y* axis) and RUNX1-IT1 RNA (*X* axis) regions. The red shading in the heatmap indicates the interaction score of a single amino acid and nucleotide pair. **e**, **f** RIP assays were performed to validate RUNX1-IT1 binding to RUNX1 in PANC-1 and SW1990 cells transfected with RUNX1-IT1 overexpression or control vectors. **g** The chart shows information about the WT and mutant RUNX1-IT1 vectors. **h** RIP assays were performed to validate RUNX1-IT1 binding to RUNX1 in PANC-1 cells transfected with RUNX1-IT1 (WT) and mutant RUNX1-IT1 overexpression vectors. **i** EdU assays were used to assess proliferation in the three groups of PANC-1 cells. **j** The migration and invasion abilities of the three groups of cells were assessed by a transwell assay. **k** Histogram showing the proliferation rates of cotransfected cells in the three groups. **l**, **m** Histogram showing the number of migrated and invaded cotransfected cells in the three groups. **n** Representative MRI images of liver metastatic tumors in the three groups of mice are shown. Images of liver surfaces and HE staining of metastatic tumor lesions in the different groups are shown (scale bars, 1000 and 100 μm). (o) Histogram indicating the numbers of metastasized lesions in the three groups of mice (*P < 0.05, **P < 0.01, ***P < 0.001).

To clarify the potential mechanisms of RUNX1-IT1 in PC cells, we examined its distribution using fluorescence in situ hybridization (FISH) and subcellular fractionation. RUNX1-IT1 was predominantly localized in the nucleus (Fig. 4a, b), and it has been suggested to play a biological role in transcription by interacting with chromatin or nuclear proteins^21^. We next calculated the interaction probabilities of RUNX1 and RUNX1-IT1 (http://pridb.gdcb.iastate.edu/RPISeq/) and found that RUNX1-IT1 potentially binds to RUNX1 (RF: 0.85, SVM: 0.78; RF or SVM scores > 0.5 were considered “positive”). To further identify the potential region of binding between RUNX1-IT1 and the transcription factor RUNX1, we used the CatRAPID online algorithm (http://service.tartaglialab.com/page/catrapid_group), which rapidly predicts RNA-protein interactions and domains to evaluate the interaction tendency based on the secondary structures, hydrogen bonding, and molecular interatomic forces^22^. CatRAPID analysis showed that the 1111 bp-1173 bp region of RUNX1-IT1 is the most likely to be bound by RUNX1 (Fig. 4c, d). Next, we performed RIP assays in PC cells transfected with RUNX1-IT1 overexpression or control vectors. The results confirmed that RUNX1 directly bound to RUNX1-IT1, and higher enrichment was observed in the overexpression group than in the control group (Fig. 4e, f). Furthermore, we constructed a mutant RUNX1-IT1 vector according to the predicted binding sites to identify the binding sites of RUNX1-IT1 and RUNX1 (Fig. 4g). The RIP assays showed minimal enrichment in the mutant RUNX1-IT1 group compared with the WT group (Fig. 4h), consistent with our prior CatRAPID prediction.

To confirm whether RUNX1-IT1 plays a biological role in PC via the transcription factor RUNX1, we first established stable PANC-1 and SW1990 cells ectopically overexpressing RUNX1-IT1 via a lentiviral infection system (Additional file 9: Supplementary Fig. S3g). Then, RUNX1-IT1-overexpressing SW1990 and PANC-1 cells were transfected with sh-control or sh-RUNX1-1 lentiviral vectors. The three groups (NC, ex-RUNX1-IT1 and ex-RUNX1-IT1 + sh-RUNX1) were subjected to EdU and transwell assays, revealing that RUNX1 partially rescued RUNX1-IT1-induced PC cell proliferation, migration and invasion (Fig. 4i–m; Additional file 11: Supplementary Fig. S5a–d). To further investigate this phenomenon in vivo, 15 mice were randomly divided into three groups and a total of 10^6^ stably transfected PANC-1 cells were injected into the distal pancreatic tissues of 4- to 6-week-old male nude mice. Tumor progression was assessed by a small-animal MRI instrument after 6–8 weeks. Notably, the ex-RUNX1-IT1 group exhibited more liver metastasis than the NC group, while the incidence of liver metastasis was reduced in the ex-RUNX1-IT1 + sh-RUNX1 group. Representative MRI images, surface metastases on mice livers and HE staining are shown in Fig. 4n, o. Taken together, these findings clearly indicate that RUNX1-IT1 regulates PC progression through RUNX1 in vitro and in vivo.

### C-FOS is a key downstream target of RUNX1-IT1 and RUNX1

**Fig. 5:**
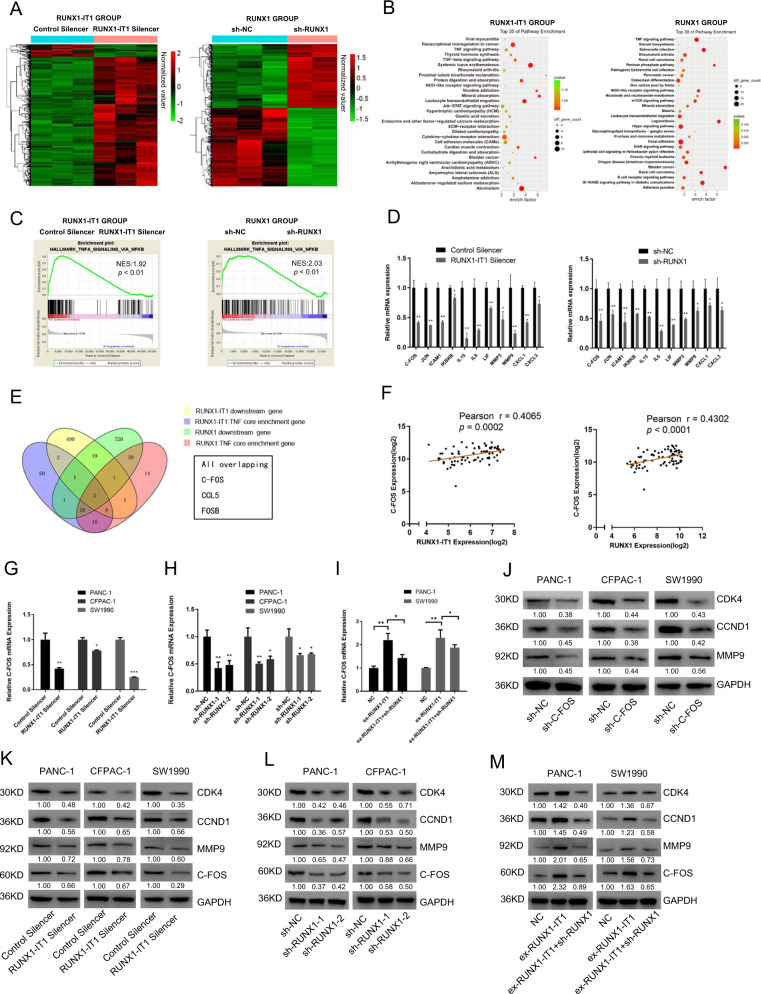
C-FOS is a critical downstream target of RUNX1-IT1 and RUNX1. **a** Heatmap of differentially expressed downstream genes in RUNX1 and RUNX1-IT1 knockdown PANC-1 cells and the corresponding control cells (*P* < 0.05, fold change > 2). **b** Top 30 enriched pathways in RUNX1 and RUNX1-IT1 knockdown PANC-1 cells. **c** GSEA analysis of the RUNX1 and RUNX1-IT1 knockdown PANC-1 groups and the control. **d** The levels of downstream TNF genes in the RUNX1 and RUNX1-IT1 knockdown PANC-1 groups were analyzed by qRT-PCR and compared with those in the control group. **e** The Venn diagram shows three targets in the area common to the four groups, namely, RUNX1-IT1 downstream genes, RUNX1 downstream genes (fold change > 2, *P* < 0.05), RUNX1-IT1 TNF core enriched genes and RUNX1 TNF core enriched genes (*P* < 0.05). **f** Correlation analysis between C-FOS and RUNX1 and between C-FOS and RUNX1-IT1 in GEO (GSE15471). **g**, **h** The C-FOS gene expression levels in the RUNX1-IT1 knockdown and RUNX1 knockdown groups were analyzed by qPCR. **i** The C-FOS gene expression levels in the NC, ex-RUNX1-IT1 and ex-RUNX1-IT1+sh-RUNX1 groups were analyzed by qRT-PCR. **j** The protein expression levels of C-FOS-related downstream molecules in the C-FOS knockdown PC and control cells were assessed by WB. **k**, **l** The protein expression levels of C-FOS and its related downstream molecules in the RUNX1-IT1 knockdown and RUNX1 knockdown groups were assessed by WB. **m** The protein expression levels of C-FOS and its related downstream molecules in the NC, ex-RUNX1-IT1 and ex-RUNX1-IT1 + sh-RUNX1 groups were assessed by WB (*P < 0.05, **P < 0.01, ***P < 0.001).

To investigate the pathway downstream of RUNX1-IT1 and RUNX1, we performed RNA-seq analyses of two PANC-1 cell groups: RUNX1-IT1 knockdown/control and RUNX1 knockdown/control. Hierarchical clustering showed that differentially expressed mRNAs perfectly distinguished RUNX1-IT1 knockdown or RUNX1 knockdown cells from the corresponding control (Fig. 5a). Pathway analysis revealed the top 30 enriched pathways in the two groups (Fig. 5b). Given that RUNX1-IT1 promotes PC cell progression through RUNX1, we selected a commonly enriched pathway for further analysis. Gene set enrichment analysis (GSEA) revealed that the TNF pathway was enriched in both the RUNX1-IT1 and RUNX1 datasets (Fig. 5c). We subsequently focused on the TNF pathway and validated the pathway regulation by qRT-PCR after knockdown of RUNX1-IT1 or RUNX1 in PANC-1 cells, and the downstream TNF genes were differentially regulated (Fig. 5d; Additional file 11: Supplementary Fig. S5e). Because the common targets produce a similar phenotype, we conducted intersection analysis of four groups: RUNX1-IT1 downstream genes, RUNX1 downstream genes, RUNX1-IT1 TNF core enriched genes and RUNX1 TNF core enriched genes, and discovered three downstream target genes, C-FOS, FOSB and CCL5 (Fig. 5e). The analysis results indicated that the three candidates were enriched in the TNF pathway and were common downstream targets of RUNX1-IT1 and RUNX1. To validate the three targets, we performed correlation analysis using PC samples in The Cancer Genome Atlas (TCGA) database and found only C-FOS was significantly correlated with both RUNX1-IT1 and RUNX1 in the clinical specimens (Additional file 12: Supplementary Fig. S6a–c). The correlation between RUNX1-IT1 or RUNX1 and C-FOS was further validated using GEO data (GSE15471, Fig. 5f). We performed qPCR assays to validate the regulatory effect and found that knockdown of RUNX1-IT1 or RUNX1 downregulated C-FOS mRNA expression in PC cells (Fig. 5g, h). Auspiciously, C-FOS is identified as a core gene downstream of TNF in the KEGG pathway database (https://www.genome.jp/kegg/pathway.html), and it participates in tumor proliferation and invasion, which could regulate CCND1, CDK4 and MMPs. Then, we performed WB analysis to detect related downstream targets in C-FOS knockdown cells (Fig. 5j; Additional file 9: Supplementary Fig. S3i). Functional assays were also performed, and the results indicated that knockdown of C-FOS dramatically inhibited cell proliferation, migration and invasion (Additional file 13: Supplementary Fig. S7a–f). C-FOS and its downstream targets were also downregulated in RUNX1-IT1 and RUNX1 knockdown cells but not in control cells, indicating the regulatory effect of RUNX1-IT1 and RUNX1 on these genes (Fig. 5k, l). Additionally, rescue experiments were performed to investigate whether RUNX1-IT1 upregulates C-FOS expression via RUNX1. Knockdown of RUNX1 reduced the mRNA and protein expression of C-FOS upregulated by RUNX1-IT1, indicating that RUNX1-IT1 upregulates C-FOS expression through the transcription factor RUNX1 (Fig. 5i, m).

**Fig. 6:**
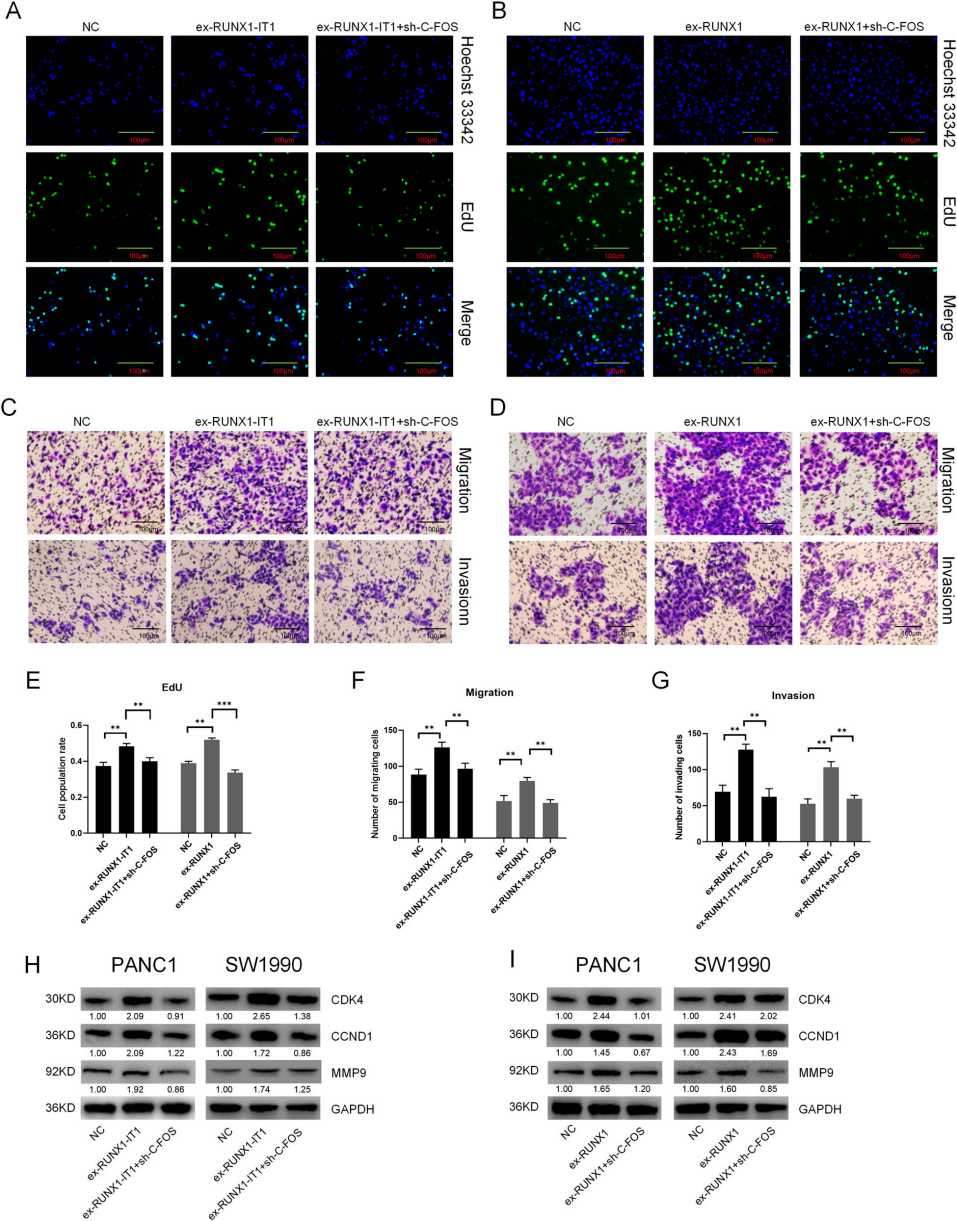
RUNX1-IT1 and RUNX1 promoted cell proliferation, migration and invasion via C-FOS. **a**, **c** Knockdown of C-FOS in RUNX-IT1-overexpressing cells. EdU and transwell assays were used to assess proliferation, migration and invasion in the NC, ex-RUNX1-IT1 and ex-RUNX1-IT1+sh-C-FOS groups of PANC-1 cells. **b**, **d** Knockdown of C-FOS in RUNX1-overexpressing cells. EdU and transwell assays were used to assess proliferation, migration and invasion in the NC, ex-RUNX1 and ex-RUNX1+sh-C-FOS groups of PANC-1 cells. **e** Histogram showing the proliferation rates of cotransfected cells. **f**, **g** Histogram showing the numbers of migrated and invaded cotransfected cells. **h** The protein expression levels of C-FOS-related downstream molecules in the NC, ex-RUNX1-IT1 and ex-RUNX1-IT1+sh-C-FOS groups were assessed by WB. **i** The protein expression levels of C-FOS-related downstream molecules in the NC, ex-RUNX1 and ex-RUNX1+sh-C-FOS groups were assessed by WB analysis (*P < 0.05, **P < 0.01, ***P < 0.001).

To further investigate whether C-FOS is an important downstream target of RUNX1-IT1 and RUNX1, we performed rescue experiments in vitro.The effect of RUNX1-IT1 or RUNX1 on PC cell proliferation, migration and invasion was partially ameliorated by C-FOS knockdown (Fig. 6a–g; Additional file 14: Supplementary Fig. S8a–e). Additionally, knockdown of C-FOS reduced the protein expression of CDK4, CCND1 and MMP9 upregulated by RUNX1-IT1 or RUNX1 (Fig. 6h, i). These findings indicate that C-FOS is a critical downstream target of RUNX1-IT1 and RUNX1.

### Increased RUNX1-IT1 and RUNX1 expression is associated with the C-FOS pathway in PC

**Fig. 7:**
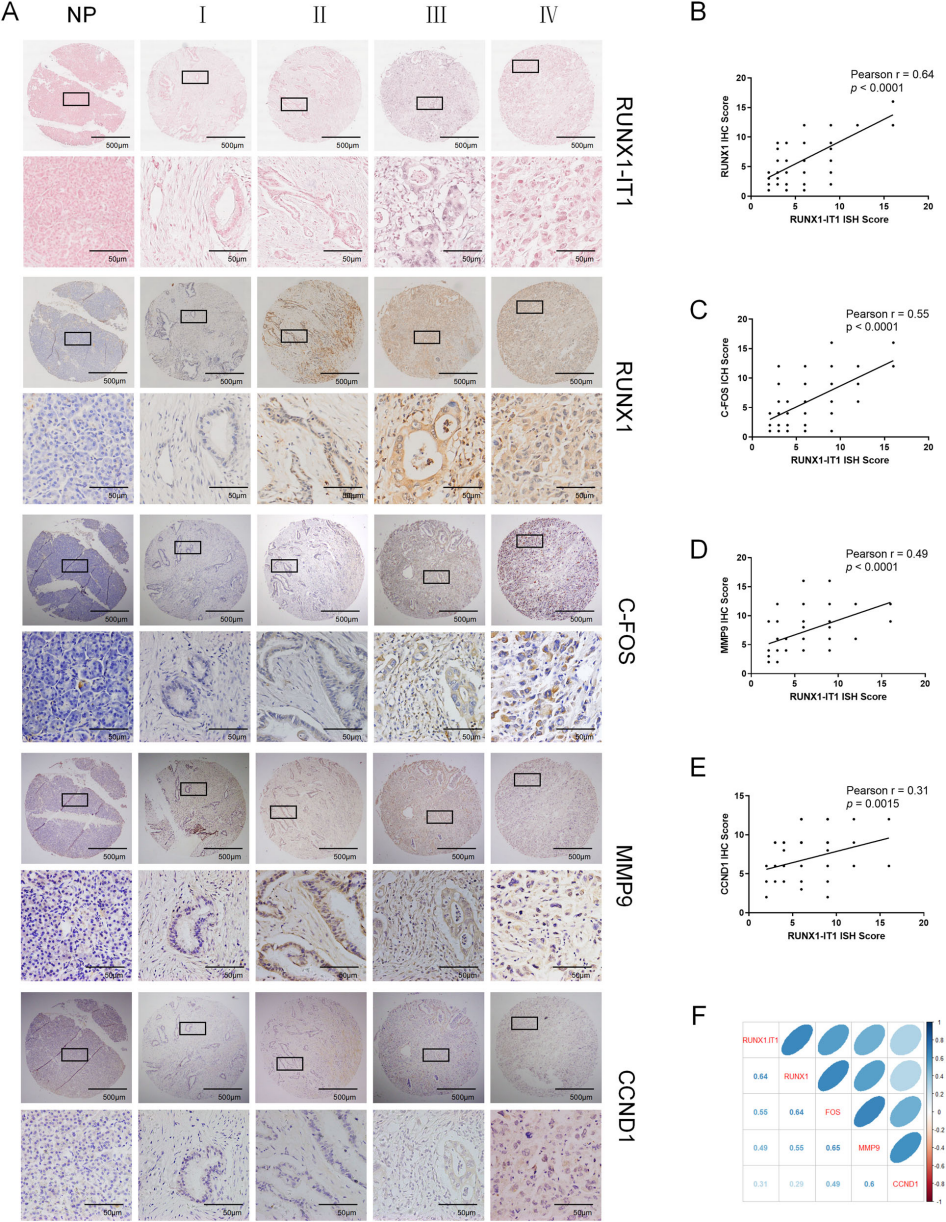
RUNX1-IT1 expression is positively associated with RUNX1 and C-FOS expression in human PC. **a** Representative images (scale bars, 500 and 50 μm) of ISH or IHC staining for RUNX1-IT1, RUNX1, C-FOS, MMP9 and CCND1 in tissues from different clinical stages (I–IV) are shown. **b**–**e** Bar charts showing the correlation between the expression of RUNX1-IT1 and RUNX1, C-FOS, MMP9 and CCND1. **f** General correlation analysis of RUNX1-IT1, RUNX1, C-FOS, MMP9 and CCND1. (*P < 0.05, **P < 0.01, ***P < 0.001).

To further assess the relationship between the expression of RUNX1-IT1 or RUNX1 and C-FOS downstream targets, we conducted IHC assays in PC tissues. Representative staining images of RUNX1-IT1, RUNX1, C-FOS, MMP9 and CCND1 in different clinical stage samples are shown in Fig. 7a. Correlation analysis indicated that RUNX1-IT1 expression was significantly correlated with that of RUNX1, C-FOS, MMP9 and CCND1 in PC samples (Fig. 7b–f).

### RUNX1-IT1 induces C-FOS expression by recruiting RUNX1 to the C-FOS promoter

**Fig. 8:**
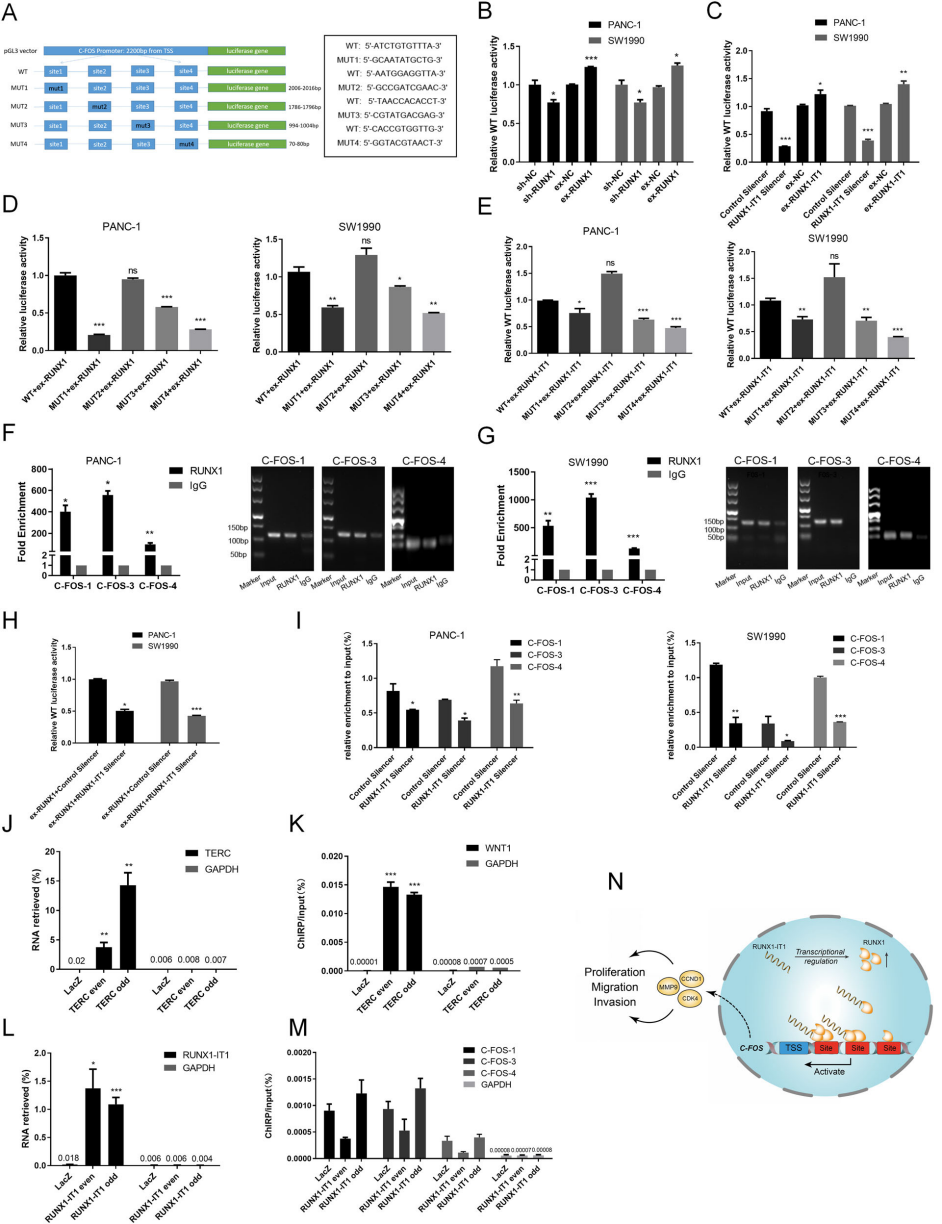
RUNX1-IT1 regulates C-FOS expression by recruiting RUNX1 to the C-FOS promoter. **a** WT and mutant C-FOS promoters were constructed using the pGL3 vector. **b**, **c** Luciferase activity assays were performed using PC cells (PANC-1 and SW1990) with RUNX1 or RUNX1-IT1 knockdown and overexpression that were cotransfected with a C-FOS WT promoter. **d**, **e** RUNX1- or RUNX1-IT1-overexpressing PC cells were cotransfected with the WT vector or with one of the MUT vectors. **f**, **g** ChIP assays with an anti-RUNX1 antibody or IgG were performed to verify the enrichment of RUNX1 at binding sites in the C-FOS promoter in PC cells. The ChIP-PCR products of the RUNX1, input and IgG groups were detected by agarose gel electrophoresis. **h** RUNX1-overexpressing PC cells were transfected with RUNX1-IT1 or Control Silencer. Luciferase activity assays were performed. **i** ChIP assays were performed in RUNX1-IT1 knockdown PC and control cells with an anti-RUNX1 antibody or IgG. **j** Retrieval of RNA by ChIRP with TERC probes (positive control). ChIRP was performed using PANC-1 cells and TERC lncRNA even, odd or NC (LacZ) probe sets. Purified RNA was then analyzed by qRT-PCR using RNA-positive control primers (TERC) and RNA-negative control primers (GAPDH). **k** Successful DNA binding by ChIRP with TERC probes. Purified DNA was then analyzed by qRT-PCR using the WNT-1 precursor (positive target) and GAPDH D2 coding region (negative target). **l** Successful retrieval of RNA by ChIRP with RUNX1-IT1 probes. **m** DNA binding by ChIRP with the RUNX1-IT1 probe. Purified DNA was analyzed by qRT-PCR using primers specific for the FOS promoter binding region and the GAPDH D2 coding region. **n** A schematic model of the mechanism underlying the role of RUNX1-IT1 in the progression of PC (*ns* no significance, *P < 0.05, **P < 0.01, ***P < 0.001).

We next addressed the mechanism by which RUNX1-IT1 contributes to C-FOS transcriptional activation. Given that RUNX1 is a transcription factor, we predicted the RUNX1 binding sites in the C-FOS promoter via bioinformatics analysis performed by Sangon Biotech and found that RUNX1 potentially binds to the promoter of C-FOS via four predicted sites (Fig. 8a). Then, we constructed the C-FOS WT promoter vector (the length of the promoter was 2200 bp from the transcription start site (TSS)) and performed reporter gene assays. Compared with the control, the group cotransfected with the C-FOS promoter vector (WT) and sh-RUNX1 vector exhibited significantly decreased luciferase activity, and the opposite pattern was observed in the RUNX1 overexpression group (Fig. 8b). Similar results were observed in the RUNX1-IT1 knockdown and overexpression groups (Fig. 8c). These results indicate RUNX1 and RUNX1-IT1 regulate C-FOS transcription via their promoters. Next, by constructing C-FOS mutant promoter vectors, we identified three obvious regulatory binding sites in PANC-1 and SW1990 cells (Fig. 8d, e). ChIP-PCR analysis revealed three differential binding sites enriched in RUNX1 (Fig. 8f, g). These data demonstrated that RUNX1 increased the activity of the C-FOS promoter reporter mainly via three binding sites.

Furthermore, to examine whether the binding of RUNX1 to the C-FOS promoter requires RUNX1-IT1, we once again performed dual luciferase reporter gene assays. C-FOS reporter gene activity was decreased in RUNX1-overexpressing cells cotransfected with the RUNX1-IT1 Silencer compared with that in the Control Silencer group, indicating that knockdown of RUNX1-IT1 attenuated the RUNX1-mediated regulation of C-FOS promoter activity (Fig. 8h). Importantly, ChIP-PCR revealed that knockdown of RUNX1-IT1 reduced the enrichment of RUNX1 in the C-FOS promoter region (Fig. 8i). These results suggest that RUNX1-IT1 activates C-FOS transcription by facilitating the binding of RUNX1 to the C-FOS promoter.

The observation prompted us to investigate whether RUNX1-IT1 also independently binds to chromatin at these sites. Using ChIRP, a technique for probing lncRNA-chromatin interactions, we isolated RUNX1-IT1-bound chromatin fragments. ChIRP-PCR revealed low RUNX1-IT1 occupancy in the C-FOS promoter region, indicating that RUNX1-IT1 could not bind directly to the sites at which it exerts a regulatory effect by affecting the ability of RUNX1 to bind these promoter regions (Fig. 8j–m). This finding confirms that RUNX1-IT1 functions as a plausible transcriptional coregulator of RUNX1. Taken together, our data indicate that RUNX1-IT1 is an important RNA component in the PC regulatory network (Fig. 8n).

## Discussion

In recent years, an abundance of sequencing data have demonstrated that many lncRNAs are dysregulated in a variety of diseases^23^, however few lncRNAs have been investigated to date. We analyzed the expression of dysregulated RNAs in PC by integrating microarrays. Through screening, we found the lncRNA RUNX1-IT1 was abnormally upregulated in PC. In vivo and in vitro experiments, we validated that RUNX1-IT1 plays an oncogenic role in PC. However, a recent study showed that RUNX1-IT1 was downregulated in colorectal cancer and inhibited tumor formation by regulating cell proliferation, migration and apoptosis^24^. These findings differed from ours presented herein, indicating that lncRNAs are complex and have diverse functions.

In general, nuclear-localized lncRNAs can play cis- and transregulatory roles by guiding and recruiting histone protein-modifying enzymes or transcription factors to specific genomic loci, leading to the inactivation of tumor suppressors or activation of oncogenes^25,26^. In our study, we first investigated the cis-regulation of RUNX1-IT1, confirming via RNA ISH that RUNX1-IT1 localizes in the nucleus and that it upregulates RUNX1 mRNA expression by activating RUNX1 transcription. Next, we demonstrated that RUNX1-IT1 specifically bound to the RUNX1 protein, which indicates that it may act as a transregulatory molecule to participate in broader regulatory functions. In recent years, an increasing numbers of studies have shown that RUNX1 acts as a tumor suppressor or cancer-promoting factor in solid tumors^17^. Studies have shown that RUNX1 is highly expressed in various tumors, such as ovarian cancer^18^, bladder cancer^27^, and colorectal cancer^28^, and promotes tumor progression through transcriptional regulation of various oncogenes. However, the mechanisms of RUNX1 regulation in PC are unclear. We demonstrated that RUNX1 acts as a cancer-promoting gene and promotes PC cell proliferation and orthotopic liver metastasis. RNA-seq analysis revealed that RUNX1 was involved in various tumor-associated pathways, such as TNF, mTOR and focal adhesions, and that its downstream targets were involved in proliferation, apoptosis, invasion and epithelial-mesenchymal transition (EMT).

Integrative analysis of the downstream regulatory network of RUNX1-IT1 and RUNX1 revealed that C-FOS may be a common target of RUNX1 and RUNX1-IT1. C-FOS is a member of the activator protein (AP)-1 complex that acts as an essential effector of other oncogenes and participates in the regulation of many processes, including proliferation, migration, apoptosis and angiogenesis^29–31^. C-FOS also contributes to PC progression and is associated with prognosis^32,33^. KEGG network analysis showed that C-FOS is an important node in the TNF signaling pathway, participating in the regulation of downstream targets such as MMP9, CCND1, CDK4. Further clinical analysis showed that the expression of C-FOS and targets was significantly associated with RUNX1-IT1/RUNX1 expression in PC tissues. These findings provide important information for clinical guidance.

In the study, we found that RUNX1-IT1 enhances the transcription of the RUNX1 gene, indicating that it may function as a cis-regulatory RNA that is involved in the upstream regulation of RUNX1; however, elucidation of the underlying mechanism requires further research. In addition, pathway and Venn diagram analyses showed numerous differences in the expression profiles of abundant downstream targets and RUNX1 and RUNX1-IT1, indicating that as a regulatory molecule, RUNX1-IT1 may have extensive biological functions in PC except for interacting with RUNX1. Interestingly enough, in addition to C-FOS, the other transcription factors regulated by RUNX1-IT1 such as CEBPA and HLF were screened using the cluster analysis (Additional file 15: Supplementary Fig. S9). Hence, further studies are necessary to address the regulatory mechanisms of other important transcription factors regulated by RUNX1-IT1 in PC.

Taken together, our results demonstrate that the lncRNA RUNX1-IT1 is an oncogenic factor that promotes PC progression by regulating and recruiting RUNX1 to the C-FOS gene promoter. The newly identified RUNX1-IT1/RUNX1/C-FOS axis may serve as a promising therapeutic target for PC.

## Supplementary information

Additional files figure legends

Additional file 1. Table S1

Additional file 2. Table S2

Additional file 3. Fig. S1

Additional file 4. Table S3

Additional file 5. Table S4

Additional file 6. Fig. S2

Additional file 7. Table S5

Additional file 8. Table S6

Additional file 9. Fig. S3

Additional file 10. Fig. S4

Additional file 11. Fig. S5

Additional file 12. Fig. S6

Additional file 13. Fig. S7

Additional file 14. Fig. S8

Additional file 15. Fig. S9

Additional file 16. Fig. S10
