# Supplementary material for: The lncRNA RUNX1-IT1 regulates C-FOS transcription by interacting with RUNX1 in the process of pancreatic cancer proliferation, migration and invasion
Source: Cell Death Dis. 2020 Jun 2;11(6):412. doi: 10.1038/s41419-020-2617-7 (PMC7265432; doi:10.1038/s41419-020-2617-7)
Supplement: Supplementary file 1 — Additional files figure legends [file 41419_2020_2617_MOESM1_ESM.docx]

**Additional files**

**Additional file 1: Table S1.** Primers used for qRT-PCR, RIP, ChIP, ChIRP and RACE, the RUNX1-IT1 sequence determined by RACE, the Smart Silencer and shRNA sequences, the antibodies used for WB and IHC, and the probes used for ISH and ChIRP.

**Additional file 2: Table S2.** Supplemental materials and methods.

**Additional file 3:** **Fig. S1.** Pancreatic cancer (PC) and normal pancreas (NP) gene microarray information. (A) Correlation analysis of PC and NP samples. (B) Principal component analysis (PCA) of PC and NP samples. PCA is usually used to evaluate the grouping of samples. (C) Volcano plots for the two groups. X-axis: Log2(fold change), Y-axis: -Log10(p-value), red area: molecules (p-value <0.05; fold change≥2), blue area: molecules (p-value <0.05, fold change≤0.5)

**Additional file 4: Table S3.** Differentially expressed genes in the three GEO datasets.

**Additional file 5: Table S4.** The 40 up-regulated genes and 23 down-regulated genes in the three GEO datasets.

**Additional file 6: Fig. S2.** RUNX1-IT1 expression was assessed by in situ hybridization (ISH) in PC samples. Images of the different levels of positive staining score are shown (scale bar, 500μm and 50μm).The staining intensity score was classified as 0 (negative), 1 (weak), 2 (moderate), 3 (moderate-strong), or 4 (strong), and the positive staining rate scores were as follows: 0 (negative), 1 (1-25%), 2 (26-50%), 3 (51-75%), and 4 (76-100%). The product of summing the "staining intensity score" and the "positive staining rate score" was considered the total ISH staining score. A total score of ≤ 4 was used for the low RUNX1-IT1 expression group, and a total score > 4 was used for the high RUNX1-IT1 expression group. **Additional file 7: Table S5.** Statistical analysis of the correlation between RUNX1-IT1 expression and clinical characteristics of PC.

**Additional file 8: Table S6.** Univariate and multivariate Cox regression analyses of the association between RUNX1-IT1 expression and survival.

**Additional file 9: Fig. S3.** (A) RUNX1-IT1 expression was analyzed by qRT-PCR in five pancreatic cancer cell lines and compared with that in a normal pancreatic cell line. (B,C) 3′and 5′ RACE analysis in PANC-1 cell; (D) qRT-PCR analysis of RUNX1-IT1 knockout and control PANC-1 cells established using the CRISP-Cas9 lentiviral system. (E) qRT-PCR analysis of PANC-1, SW1990 and CFPAC-1 cells with RUNX1 knockdown using a lentiviral system. (F) WB analysis of PANC-1, SW1990 and CFPAC-1 cells with RUNX1 knockdown using a lentiviral system. (G) qRT-PCR analysis of RUNX1-IT1-overexpressing PANC-1 and SW1990 cells using a lentiviral system. (H) qRT-PCR analysis of RUNX1-overexpressing PANC-1 and SW1990 cells using a lentiviral system. (I) WB analysis of PANC-1, SW1990 and CFPAC-1 cells with C-FOS knockdown using a lentiviral system. (*P < 0.05, **P < 0.01, ***P < 0.001).

**Additional file 10: Fig. S4.** RUNX1-IT1 upregulated the H3K27Ac level of RUNX1 gene proximal promoter. (A)The H3K27 acetylation of RUNX1 proximal promoter region in UCSC genome database.(B) ChIP assays with acetylated H3k27 specific antibody or control IgG were performed in PC cells. (C) ChIP assays with acetylated H3K27 specific antibody or control IgG were performed in RUNX1-IT1 knockdown PC cells compared with that in control cells. (*P < 0.05, **P < 0.01, ***P < 0.001).

**Additional file 11: Fig. S5.** RUNX1-IT1 functions via the transcription factor RUNX1 in SW1990 cell. (A) EdU assays were used to assess proliferation in the three groups of SW1990 cells. (B) The migration and invasion abilities of the three groups of cells were assessed by transwell assays. (C) Histogram showing the cell proliferation rates of three groups. (D) Histogram showing the number of cell migration and invasion of three groups. (E) The downstream TNF genes were analyzed by qRT-PCR in the RUNX1-IT1 or RUNX1 knockdown SW1990 cell groups compared with the control groups. (*P < 0.05, **P < 0.01, ***P < 0.001).

**Additional file 12: Fig. S6.** Correlation analysis between RUNX1 and C-FOS, FOSB or CCL5 using TCGA data. (A) Correlation analysis between RUNX1-IT1 and C-FOS and between RUNX1 and C-FOS. (B) Correlation analysis between RUNX1-IT1 and CCL5 and between RUNX1 and CCL5. (C) Correlation analysis between RUNX1-IT1 and FOSB and between RUNX1 and FOSB.

**Additional file 13: Fig. S7.** Knockdown of C-FOS significantly inhibited PC cell proliferation, migration and invasion in PC cells. (A and B) EdU assays were used to assess the cell proliferation ability in C-FOS knockdown PC cells and control. (C and D) Transwell assays were used to assess the cell migration and invasion abilities in C-FOS knockdown PC cells and control. (E) Histogram showing the proliferation rates of transfected cells. (F) Histogram showing the number of migrated and invaded transfected cells. (*P < 0.05, **P < 0.01, ***P < 0.001).

**Additional file 14: Fig. S8.** RUNX1-IT1 and RUNX1 promoted cell proliferation, migration and invasion via C-FOS in SW1990 cell. (A and C) Knockdown of C-FOS in RUNX-IT1 overexpression sw1990 cell. The EdU and transwell assays were used to assess proliferation ,migration and invasion in the three groups of SW1990 cells. (B and D) Knockdown of C-FOS in RUNX1 overexpression cells. The EdU and transwell assays were used to assess proliferation, migration and invasion in the three groups of SW1990 cells. (E) Histogram showing the proliferation rates of cotransfected cells. (F and G) Histogram showing the number of migrated and invaded cotransfected cells. (*P < 0.05, **P < 0.01, ***P < 0.001).

**Additional file 15: Fig. S9.** Heatmap of differentially expressed transcriptional factors in RUNX1-IT1 knockdown PANC-1 cells and control cells (P<0.05, fold change>2).

**Additional file 16: Fig. S10.** The English language in the manuscript has been revised by American Journal Experts (AJE). The editing certificate was shown.
