# Supplementary material for: The lncRNA RUNX1-IT1 regulates C-FOS transcription by interacting with RUNX1 in the process of pancreatic cancer proliferation, migration and invasion
Source: Cell Death Dis. 2020 Jun 2;11(6):412. doi: 10.1038/s41419-020-2617-7 (PMC7265432; doi:10.1038/s41419-020-2617-7)
Supplement: Supplementary file 2 — Additional file 1. Table S1 [file 41419_2020_2617_MOESM2_ESM.docx]

| **Supplementary Table S1** |  |  |
| --- | --- | --- |
| **qRT-PCR primer name** | **primer sequence (5’-3’)** |  |
| GAPDH (Forward) | CAGGAGGCATTGCTGATGAT |  |
| GAPDH (Reverse) | GAAGGCTGGGGCTCATTT |  |
| β-actin (Forward) | CCTGGCACCCAGCACAAT |  |
| β-actin (Reverse) | GGGCCGGACTCGTCATAC |  |
| RUNX1-IT1 (Forward) | ACCACCTTGGTTAAGCACCC |  |
| RUNX1-IT1 (Reverse) | CACATTGGCTGTTGTGGTGG |  |
| RUNX1 (Forward) | GCCCATCGCTTTCAAGGT |  |
| RUNX1(Reverse) | AGCTCAGCCGAGGTAGTTTTCAT |  |
| FOS(Forward) | AGAATCCGAAGGGAAAGGAA |  |
| FOS (Reverse) | CTTCTCCTTCAGCAGGTTGG |  |
| MMP3(Forward) | AAGCTGGACTCCGACACTCTGG |  |
| MMP3 (Reverse) | CCTCCACTTCGGGATGCCAG |  |
| MMP9 (Forward) | CCCTTGTGCTCTTCCCTGGA |  |
| MMP9 (Reverse) | TCTGCCACCCGAGTGTAACC |  |
| ICAM1 (Forward) | GGAACAACCGGAAGGTGTATG |  |
| ICAM1(Reverse) | TGCCAGTTCCACCCGTTCT |  |
| IKKB(Forward) | TGAGGATGAGAAGACTGTTGTC |  |
| IKKB (Reverse) | TCCTGTATGGCATTTTCTAGCA |  |
| IL6 (Forward) | ATGAGGAGACTTGCCTGGTGAA |  |
| IL6 (Reverse) | GTTGGGTCAGGGGTGGTTATT |  |
| IL15 (Forward) | GGAAAGTGATGTTCACCCCAG |  |
| IL15 (Reverse) | CCTCACATTCTTTGCATCCAGAT |  |
| CXCL1 (Forward) | GATCATTGTGAAGGCAGGGGA |  |
| CXCL1(Reverse) | TAAGCCCCTTTGTTCTAAGCCAG |  |
| CXCL2(Forward) | AGATCAATGTGACGGCAGGG |  |
| CXCL2(Forward) | TCTCTGCTCTAACACAGAGGGA |  |
| CXCL3 (Forward) | CCAAACCGAAGTCATAGCCACA |  |
| CXCL3 (Forward) | TGGTGCTCCCCTTGTTCAGTAT |  |
| JUN (Forward) | AGAGCGGACCTTATGGCTACAG |  |
| JUN (Forward) | TCTCGCCTGGAATACCGATGTC |  |
| U6 (Forward) | CTCGCTTCGGCAGCACA |  |
| U6 (Reverse) | TCTCGCCTGGAATACCGATGTC |  |
|  |  |  |
| **3’5’-RACE-PCR primer name** | **primer sequence (5’-3’)** |  |
| 3’adapte | GCTGTCAACGATACGCTACGTAACGGCATGACAGTGTTTTTTTTTTTTTTTTTT |  |
| 5.3’ outer | GCTGTCAACGATACGCTACGTAAC |  |
| 5.3’ inner | GCTACGTAACGGCATGACAGTG |  |
| RUNX1-IT1 (RC393-F3) | CGGAGCCCCAAGTTTCTCATCTGTAA |  |
| RUNX1-IT1 (RC393-F4) | AAGGGGAATGAGCCCTACTTTGTATGGTT |  |
| **5’-RACE-PCR primer name** |  |  |
| 5’adapter | GCTGTCAACGATACGCTACGTAACGGCATGACAGTGCCCCCCCCCCCCCCC |  |
| RUNX1-IT1 RC393-R1 | TTTCTCGTTCGGACTCTGGCACCAA |  |
| RUNX1-IT1 RC393-R2 | CTATGGAACCCTGGGCAAGTGAACCT |  |
| RC393-RT2 | CATTGCCTAAGGAGCCA |  |
| RC393-RT1 | GCCCTAGACCCTCATCA |  |
|  |  |  |
| **RIP qRT-PCR primer name** | **primer sequence (5’-3’)** |  |
| RUNX1-IT1 (Forward) | ACCACCTTGGTTAAGCACCC |  |
| RUNX1-IT1 (Reverse) | CACATTGGCTGTTGTGGTGG |  |
|  |  |  |
| **CHIP qRT-PCR primer name** | **primer sequence (5’-3’)** |  |
| FOS-1F (Forward) | CTTTAGGATTCAGAGCAGCTCCA |  |
| FOS-1R (Reverse) | TGTTGGTGGGAATTTAGGCG |  |
| FOS-4F (Forward) | TGTCATCCCGAACTGACCAC |  |
| FOS-4R (Reverse) | CTGTGCAAAACCTACGTGCG |  |
| RUNX1 H3K27Ac (Forward) | ACCCATTACAGAAACCGACCC |  |
| RUNX1H3K27Ac (Reverse) | ATTCCTTGCATGAGGCCGGA |  |
|  |  |  |
| **CHIRP qRT-PCR primer name** | **primer sequence (5’-3’)** |  |
| RNA Positive Control , TERC (Forward) | CGCTGTTTTTCTCGCTGACT |  |
| RNA Positive Control, TERC (Reverse) | GCTCTAGAATGAACGGTGGAA |  |
| RNA Negative Control, GAPDH (Forward) | GTCGGAGTCAACGGATTT G |  |
| RNA Negative Control, GAPDH (Reverse) | TGGGTGGAATCATATTGGAA |  |
| RUNX1-IT1 (Forward) | ACCACCTTGGTTAAGCACCC |  |
| RUNX1-IT1 (Reverse) | CACATTGGCTGTTGTGGTGG |  |
| ChIRP WNT-1 precursor (Forward) | AGGGCTGGAATTTCAAAGGT |  |
| ChIRP WNT-1 precursor (Reverse) | TTCTCCTCAGGATGTACCCG |  |
| CHIRP GAPDH coding D2 (Forward) | GGCTCCCACCTTTCTCATCC |  |
| CHIRP GAPDH coding D2 (Reverse) | GGCCATCCACAGTCTGG |  |
| ChIRP FOS-1F (Forward) | CTTTAGGATTCAGAGCAGCTCCA |  |
| ChIRP FOS-1R (Reverse) | TGTTGGTGGGAATTTAGGCG |  |
| ChIRP FOS-4F (Forward) | TGTCATCCCGAACTGACCAC |  |
| ChIRP FOS-4R (Reverse) | CTGTGCAAAACCTACGTGCG |  |
|  |  |  |
|  |  |  |
| **si or sh-RNA sequence** |  |  |
| **RUNX1-IT1 SMART SILENCER** | **siRNA sequence (5’-3’)** |  |
| SMART SILENCER1 | CCATTCAAGCATGCACATA |  |
| SMART SILENCER2 | GCTCAGAAGTGGCCTCTCA |  |
| SMART SILENCER3 | CTCCTTAGGCAATGTTACA |  |
| SMART SILENCER4 | CCTGGGAAGTGGGAGATCTT |  |
| SMART SILENCER5 | GGTTGGTGCCAGAGTCCGAA |  |
| SMART SILENCER6 | GCACATCCATGCAGAGGGTT |  |
| **shRNA name** | **target sequence (5’-3’)** |  |
| shRNA-RUNX1-1 | TCGAAGACATCGGCAGAAA |  |
| shRNA-RUNX1-2 | ACCACTCCACTGCCTTTAA |  |
| shRNA NC | TTCTCCGAACGTGTCACGT |  |
| shRNA C-FOS | GGAGACAGACCAACTAGAA |  |
| **Crisper Cas9** | **target sequence (5’-3’)** |  |
| sgRNA1 | GTTCCTCTCCTC TAGAACTT |  |
|  | TTGAGCATATG CTATGCGCA |  |
| sgRNA2 | GATATGGAATG ATTGACAG |  |
|  | GCACTGCTACA TGATGAGAG |  |
| nc | CGCTTCCGCGGCCCGTTCAA |  |
|  |  |  |
| **Antibody** |  |  |
| RUNX1 （WB/ICH/CHIP/RIP） | Abcam（ab35962，ab23980） |  |
| FOS（WB） | Proteintech (66590-1-Ig) |  |
| FOS（ICH） | Sigma（AB1584） |  |
| CCND1（WB/ICH） | Cell Signaling Technologies( 55506S) |  |
| MMP9（WB/ICH） | Proteintech(10375-2-AP) |  |
| CDK4（WB/ICH） | Cell Signaling Technologies(12790S) |  |
| GAPDH（WB） | Proteintech（60004-1-Ig） |  |
| H3K27Ac（CHIP） | Cell Signaling Technologies（8173S） |  |
|  |  |  |
| **PROBE** |  |  |
| **ISH PROBE** | **probe sequence (5’-3’)** |  |
| RUNX1-IT1 | AGTGTCTGCTAGTTATGTGCAT-/3,5cy3/ |  |
|  | AGTGTCTGCTAGTTATGTGCAT-/3,5dig/ |  |
|  |  |  |
| **CHIRP probe RUNX1-IT1**  (RiboTM) | **probe sequence (5’-3’)** |  |
| h-RUNX1-IT1(3bio)_ChIRP Probe_1 | ATATTCGGCTCTTCCTTTCT-/3bio/ |  |
| h-RUNX1-IT1(3bio)_ChIRP Probe_2 | GATTAGGAGAAGTGTCTGCT-/3bio/ |  |
| h-RUNX1-IT1(3bio)_ChIRP Probe_3 | CTGCCTAATGAGTTCACAAG-/3bio/ |  |
| h-RUNX1-IT1(3bio)_ChIRP Probe_4 | AACATCCAAATGACAGCAGG-/3bio/ |  |
| h-RUNX1-IT1(3bio)_ChIRP Probe_5 | TGTAACATTGCCTAAGGAGC-/3bio/ |  |
| h-RUNX1-IT1(3bio)_ChIRP Probe_6 | CAGGAGTGATGTGAGCATAT-/3bio/ |  |
| h-RUNX1-IT1(3bio)_ChIRP Probe_7 | CCAATGGATTCCCTAAGCAT-/3bio/ |  |
| h-RUNX1-IT1(3bio)_ChIRP Probe_8 | AAAGGTCTGTAGAGAGAAGC-/3bio/ |  |
| h-RUNX1-IT1(3bio)_ChIRP Probe_9 | AAGGCAACTCGTAGAAGAAG-/3bio/ |  |
| h-RUNX1-IT1(3bio)_ChIRP Probe_10 | TGACAGGGAGAAACCAATAC-/3bio/ |  |
| h-RUNX1-IT1(3bio)_ChIRP Probe_11 | CTTATTGACTTCCTCTGCGT-/3bio/ |  |
|  |  |  |
| **RUNX1-IT1 sequence** |  |  |

AGGAAGAGCCGAATATTTGGCTGGAGTGATTTTTAGACATTTCTCTACCTTAGGGCACCAGAGAGGTTCACTTGCCCAGGGTTCCATAGCTGGTTGGTGCCAGAGTCCGAACGAGAAACCAGGTCTTCTCAGCTCCTGTCACCCACAGTTTTGCAGACACTGAATTTATGAGGTGGATTCCATTCAAGCATGCACATAACTAGCAGACACTTCTCCTAATCCTCCTCAATTCCCCTTTGCATTGCTCAGATACCACAATCCTTTGCAGGTTAAATCCCTTCACCTTGTGAACTCATTAGGCAGGGCAATGTATATGAATCCCTGAGTCCTTAAGAACGTCCTTAGGCTGTGTTAGAAATGCAGTTGCCATAGGAGTAACCGGCCCCTGCTGTCATTTGGATGTTCTCTCTTCCCCAAAATCCAGCACCACCTTGGTTAAGCACCCACCATGTGCCTGCCCACCTGGCTCCTTAGGCAATGTTACAATTAACCAGGTTGGTTTTGATGAGGGTCTAGGGCAAAATTTGAACCACCACAACAGCCAATGTGCACATCCATGCAGAGGGTTCAGAGATGGCCTCGGCCAAGGCTGCCAGGCTTCAGCTCTGGAAGGAGCCCTGGGAAGTGGGAGATCTTTGTATAAAAATTGGAACCCAAACTATAATTCCCATTAGGGATATATATGCTCACATCACTCCTGATTCAAAGCTCAGAAGTGGCCTCTCAGGAGAAATAAAGGTCTCTCTCTCTCTCTCTCTCTATCTCACACACACACACACACACACACACACACACACAGAGAGAGAGAGAGAGAGAGAGAGAGAGAGAGAGAGAGAGAGGCCAGCATTCAAAATTCCCATGCTTAGGGAATCCATTGGGACTTCTCCCCAGGATGTACTGAATTCAAGGAAGCTTTCTCTAGGTGTAGCAGAAACTGCTGCTGTCATGTCTCTGCTCACCAGGACGTAGCTTCTCTCTACAGACCTTTATTTCTTTCCCTGGAGGCTTCAGTCCATGTTGAAGTGTAAACTCCACTCAGCTCCAGGAGGAATCGTGTTTTCTTTATCACCAGGGGCTTCTTCTACGAGTTGCCTTTGATAGGGAGGCCAGGAGGAAGATAGGCCCAAGCTCAGGGGTGGGATCGGGGAGCAGGAAGCCTGTGGGCTTTAGAATCGAGGTATTGGTTTCTCCCTGTCACCATCATCCACCACCTGTGTGAACTTGAGCCATTTATCGAACCTCACGGAGCCCCAAGTTTCTCATCTGTAAACAAGGGGAATGAGCCCTACTTTGTATGGTTGTCAAGAGGATTTGAGACAATATGTATAAAGCAATGGACACGCAGAGGAAGTCAATAAGTACAAGGTAACTCTGAAAATGCCACCAAAGGGAGGCTAGGGACAGGAAAGCCATCTCCGCCAACCTCAAGAACGTGGCCCCGAAGCTGTTCCAGGAACTGGGCATGTATGAAGATAAAAAAAAAAAAAAAAAA
