# Supplementary material for: The lncRNA RUNX1-IT1 regulates C-FOS transcription by interacting with RUNX1 in the process of pancreatic cancer proliferation, migration and invasion
Source: Cell Death Dis. 2020 Jun 2;11(6):412. doi: 10.1038/s41419-020-2617-7 (PMC7265432; doi:10.1038/s41419-020-2617-7)
Supplement: Supplementary file 3 — Additional file 2. Table S2 [file 41419_2020_2617_MOESM3_ESM.docx]

**Additional file 3. Table S2**

**Supplemental Materials and Methods**

***Microarray data analysis***

LncRNAs were analyzed using GeneChip Human Transcriptome Array 2.0, and all data were uploaded to the Gene Expression Omnibus (GEO, GSE132956).The entries are scheduled to be released on Jun 18, 2022. According to the correlation analysis and principal component analysis (PCA), the consistency of the N1 sample was poor, and it was thus excluded (Additional file 3: Fig. S1). Two other independent datasets downloaded from GEO (GSE15471 and GSE16515) were analyzed in this study. After log^2^ transformation, the original normalized signaling data were processed using the R package pheatmap to produce a heatmap.

***RNA sequencing analysis***

PANC-1 cells were transfected with RUNX1-IT1 and control Smart Silencer and with sh-RUNX1 and control lentiviral vectors. Total RNA was extracted from the samples using the TRIzol method, analyzed by a NanoDrop 2000, and denatured by gel electrophoresis. RNA-seq was performed by SHBIO (Shanghai, China) and Novogene (Beijing, China). All the sequencing samples were transfected three times in biological replicates, and the transfection efficiency was found to be low for only one shRUNX1 construct in the subsequent analysis; this construct was thus ruled out. The complete raw data were uploaded to GEO (GSE135783). All the original datas would be scheduled to be released on August 13, 2022.

***Luciferase reporter assays***

The RUNX1-binding motif in the promoter region of C-FOS was predicted by Sangon Biotech. The RUNX1 promoter sequence and the C-FOS wild-type (WT) and mutant sequences were synthesized and then inserted into the pGL3-Basic vector (Sangon, Shanghai, China). All vectors were verified by sequencing, and luciferase activities were assessed using a Dual Luciferase Assay kit (Promega, USA) according to the manufacturer's instructions.

***Analysis by 5’ and 3’ rapid amplification of cDNA ends***

Total RNA was isolated with TRIzol reagent (Invitrogen, USA) according to the manufacturer's instructions. 3′ and 5′ RACE was performed by Sangon Biotech (Shanghai, China). The first-strand cDNA synthesis was performed with an M-MuLV Reverse Transcriptase kit (Sangon Biotech), and the PCRs were performed using LA Taq (TaKaRa, Japan). The primers are listed in Additional file 1: Table S1.

***PC cell transfection***

CFPAC-1, PANC-1 and SW1990 cells were used for RUNX1-IT1 knockdown experiments with Smart Silencer lncRNA (RiboTM h-RUNX1-IT1 Smart Silencer, Guangzhou, China). PC cells were transfected using Lipofectamine 3000 (Invitrogen, USA) following the manufacturer's protocol. Wild-type RUNX1-IT1 and mutant lentiviral vectors were constructed by GenePharma Company (Shanghai, China). The RUNX1-IT1 Crisper Cas9, RUNX1 overexpression and interference lentiviruses were constructed by GeneChem (Shanghai, China). Lentiviral infection was performed according to the manufacturer's instructions. The shRNAs targeting RUNX1 (GenBank accession no. NM_001001890) were sh-RUNX1-#1 and sh-RUNX1-#2. All sequences are shown in Additional file 1: Table S1

***RNA extraction and quantitative real-time PCR***

Total RNA was extracted from tissues or cells using an Ultrapure RNA kit (Cwbio, China). The RNA concentration was measured with a NanoDrop ND-2000 spectrophotometer (Life Technologies). Reverse transcription and real-time PCR was performed using a PrimeScript RT reagent kit and SYBR® Premix Ex Taq™ kit (Takara, Japan) according to the manufacturer's protocol. Relative mRNA expression levels were calculated using the 2-ΔΔCt method and normalized to GAPDH expression levels. All primer pairs are shown in Additional file 1: Table S1

***Western blot analysis***

Cells were lysed in cold, freshly prepared RIPA buffer (Sigma, USA) and measured with a BCA Protein Assay kit (Beyotime, China). The protein samples (30 μg) were separated by 4%-20% gradient SDS-polyacrylamide gel electrophoresis (PAGE, GenScript, USA) and transferred to polyvinylidene fluoride (PVDF, Millipore, USA) membranes for immunoblotting. The membranes were hybridized with a primary antibody at 4°C overnight followed by incubation with a secondary antibody for 1 h at room temperature. Horseradish peroxidase-conjugated secondary antibodies (Cell Signaling Technology) and an ECL chemiluminescence kit (GE, USA) were used to detect bound antibodies. Antibodies were as described in Additional file 1: Table S1

***Immunohistochemical analysis***

Patient pathological tissues were made into a tissue array, including 105 PC and 13 normal tissues. After dewaxing and hydration, the tissue arrays were washed three times with PBS. Antigen retrieval was performed by the high-pressure method for 2-3 min in citrate buffer (pH 6.0). After natural cooling, the array was incubated in 3% H2O2 for 10 min and blocked in 10% goat serum at room temperature for 15 min. Then, the arrays were incubated with primary antibody at 4℃ overnight. An anti-mouse/rabbit immunohistochemical detection kit (ZSGB-Bio, China) was used to detect the immunohistochemical reaction. Finally, the arrays were dehydrated, stained with hematoxylin and mounted with neutral resin. Antibodies were as described in Additional file 1: Table S1.

***Fluorescence in situ hybridization (FISH) and subcellular fractionation***

PC cells were fixed in formaldehyde, permeabilized with Triton X-100, and dehydrated with ethanol. The dried cells were mixed with 1 µg/µl of the FISH probe (RUNX1-IT1 sequence: AGTGTCTGCTAGTTATGTGCAT) in a hybridization buffer and incubated at 37℃ overnight. The following day, the slides were washed, dehydrated, and nuclei were finally stained using DAPI. Staining was performed, avoiding light, for 20 min, and cells were observed under a fluorescence microscope. Cytoplasmic and nuclear fractions were collected from PC cells as described in the Nuclear/Cytoplasmic Isolation kit protocol (Biovision, Milpitas, CA, USA)

***RNA in situ hybridization (ISH)***

For tissue microarray hybridization, the lncRNA RUNX1-IT1 probe was labeled with digoxin (Exiqon, Denmark). For PC cell lines, the RUNX1-IT1 probe was labeled with cyanine-3 (GenePharma, China).

***Digoxin in situ hybridization***

The lncRNA RUNX1-IT1 probe was labeled with digoxin (Exiqon, Denmark) for tissue hybridization in accordance with the manufacturer’s instructions. The pancreatic tissue array was dewaxed and hydrated and then washed with PBS. The array was incubated with 15 µg/ml protease K at 37°C for 40 min. After washing with PBS, the array was dehydrated through a gradient of 70%, 96%, and 100% ethanol. Then, 50-100 µl of hybridization solution was added to each array. The array was covered on 22 × 22 glass and hybridized for 1 h at 50℃. Then, the array was washed with 5× SSC, 1× SSC, and 0.2× SSC at 50℃ and washed with PBS at room temperature. After washing the array was placed into the sealing solution and sealed for 15 min (1 ml sealing solution: 10× Roche sealing solution 100 µl/1× maleic acid buffer 900 µl). The sealant was dried with paper and incubated with probe fragments (1:800) overnight at 4°C. After incubation, the slide was washed with TBST. The reaction NBT/BCIP reaction was incubated for 1 h in a wet box in the dark (diluted NBT/BCIP buffer: 20 µl Roche reagent buffer/1 ml NBT/BCIP diluent). The slide was washed with TBST, and 200 µl of red nuclear fixation dye was added for 1 min. Then, the slide was placed under running water for 10 min and dehydrated with alcohol. The slide was sealed with glycerin buffer.

***RNA immunoprecipitation***

RIP was performed using a Magna RIP™ RNA-Binding Protein Immunoprecipitation kit (Merck Millipore, Germany) according to the manufacturer’s instructions. PANC-1 and SW1990 cells were lysed in complete RIP lysis buffer, and the cell extracts were incubated with magnetic beads conjugated to RUNX1 antibody or control IgG for 12 h at 4℃. Beads were washed and incubated with proteinase K to remove proteins. Finally, purified RNA was subjected to qRT-PCR analysis. The antibodies used in RIP included RUNX1 (Abcam, Cambridge, MA) and IgG (Merck Millipore, Germany).

***Chromatin immunoprecipitation***

ChIP experiments were performed using a MagnaChIP kit (Millipore) according to the manufacturer's instructions. For the ChIP experiment, PC cells were crosslinked with 1% formaldehyde, and the reaction was quenched by the addition of 125 mM glycine. Then, the cells were washed and lysed with cell lysis buffer, and chromatin was sheared to fragments of 100-500 bp by sonication for seven cycles at high amplitude (cycles of 15 s on followed by 45 s off). Then, 5 µg of rabbit anti-RUNX1 or rabbit IgG control antibody was adsorbed onto protein G magnetic beads and incubated with the chromatin extracts at 4°C overnight. Crosslinking of DNA fragments was reversed by ChIP elution buffer and subsequent incubation at 62°C for 2 h and 95°C for 15 min. Recovered DNA was subjected to RNase treatment and analyzed via qPCR. Sequences of primers used for the ChIP-qPCR quantification are provided in Additional file 1: Table S1.

***Chromatin isolation by RNA purification***

ChIRP was performed using a Magna ChIRP RNA Interactome kit (Millipore, USA) according to the manufacturer's instructions. The sequences for the 3’-biotin-labeled probes are provided by commercial sources (RiboBio, Guangzhou, China). The recovered RNA and DNA was analyzed by qPCR. Sequences of probes and primers used for the ChIRP-qPCR quantification are provided in Additional file 1: Table S1.

***Cell proliferation assay***

For the cell proliferation assay, a total of approximately 2500 PC cells were plated in 96-well plates. After 24 h of culture, cell proliferation was assessed using CCK8 (Dojinodo, Shanghai, China) according to the manufacturer’s instructions. An EDU assay was performed to evaluate cell proliferation using a Cell Light™ EDU kit (RiboBio, Guangzhou, China) according to the manufacturer’s instructions. All experiments were performed in triplicate.

***Wound healing assay***

PC cells were seeded in a 6-well plate and incubated at 37°C until the cell confluence reached 100%. A linear wound was made in the monolayer with a pipette tip. After washing with PBS three times, the cells were incubated in a cell incubator with serum-free DMEM. Cell migration across the wound was measured by photographing the wound after 48 h. Three independent replicates of each experiment were performed.

***Transwell assay***

For migration assays, cells (5×104) were suspended in 100 µl of serum-free DMEM and seeded in the top chamber of the transwell (8 μm, 24-well format, Millipore, USA). Then, complete medium with serum (500 µl) was added to the bottom chamber. Before invasion assays, the transwell chambers were coated with 30 μl of basement membrane Matrigel (diluted 1:4 in DMEM, Corning Life Science, USA) for 5 h in a 37° incubator, and 1×105 cells were seeded in the top chamber. After a 24 h incubation, the chambers were fixed with 4% paraformaldehyde for 30 min and stained with crystal violet (Beyotime, China). Photographs of three randomly selected fields of fixed cells were captured, and the cells in the imaged fields were counted. Three independent replicates of each experiment were performed.

***Animal experiments***

Four- to six-week-old male athymic nude mice were obtained from Daping Hospital (Chongqing, China). The conditions and methods for our animal experiments were carried out in aseptic conditions. Accordingly, after a midline incision of the anterior abdominal wall, 1×106 cells (in a total volume of 0.1 ml PBS) were directly injected into the pancreas parenchyma. Anesthesia was by pentobarbital sodium. After 6 weeks, a 7.0 T small animal MRI (Bruker Biospec, Germany) was used to scan the mice to observe tumor metastasis. All efforts were made to minimize suffering, and all mice were sacrificed after the procedure. Finally, the sections were stained with HE. All animal experiments were approved by the Institutional Animal Care and Use Committee of Southwest Hospital, Chongqing, China.
