# Supplementary figures and images for: The lncRNA RUNX1-IT1 regulates C-FOS transcription by interacting with RUNX1 in the process of pancreatic cancer proliferation, migration and invasion
Source: Cell Death Dis. 2020 Jun 2;11(6):412. doi: 10.1038/s41419-020-2617-7 (PMC7265432; doi:10.1038/s41419-020-2617-7)

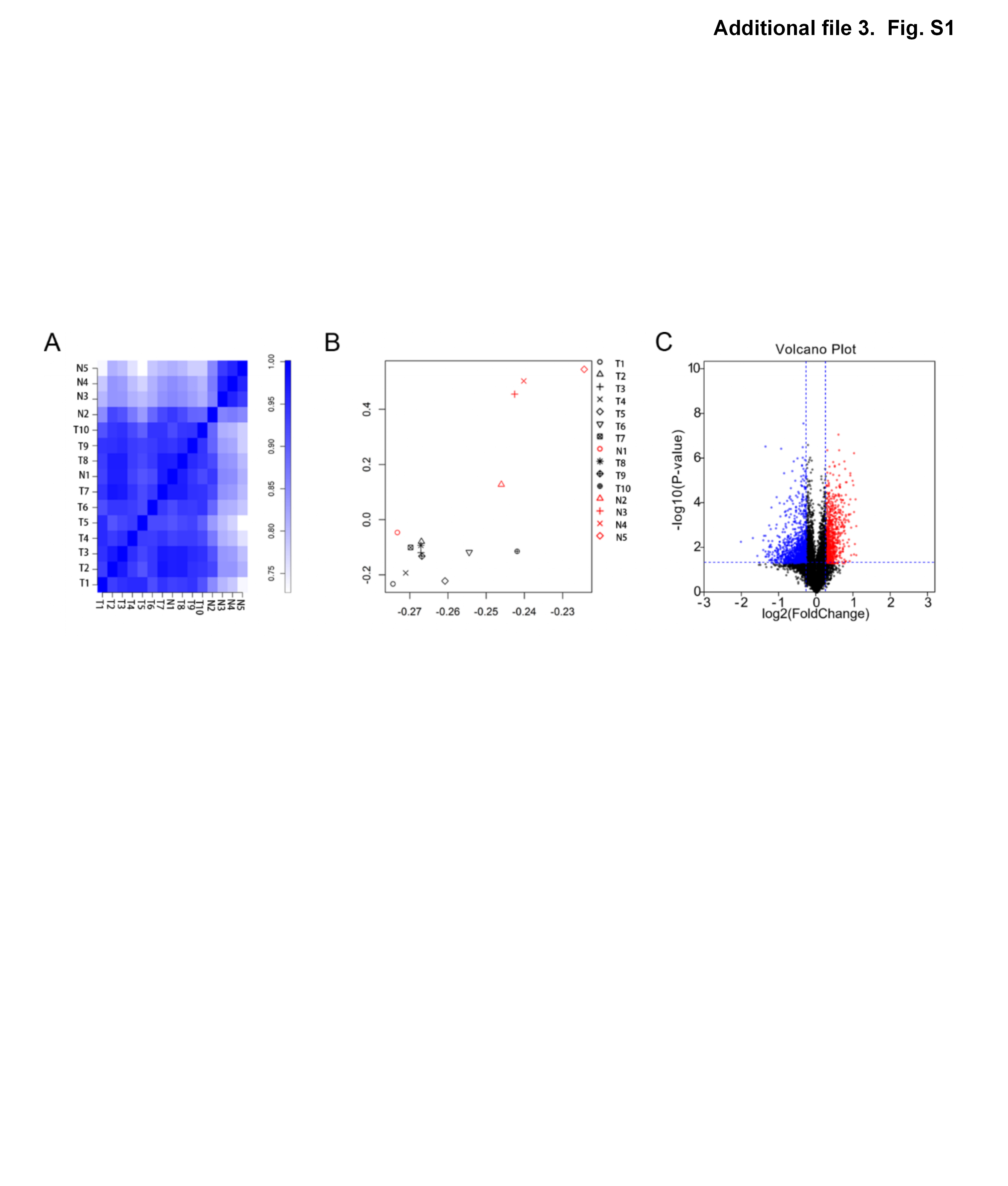

Supplement: Supplementary file 4 — Additional file 3. Fig. S1 [file 41419_2020_2617_MOESM4_ESM.tif]

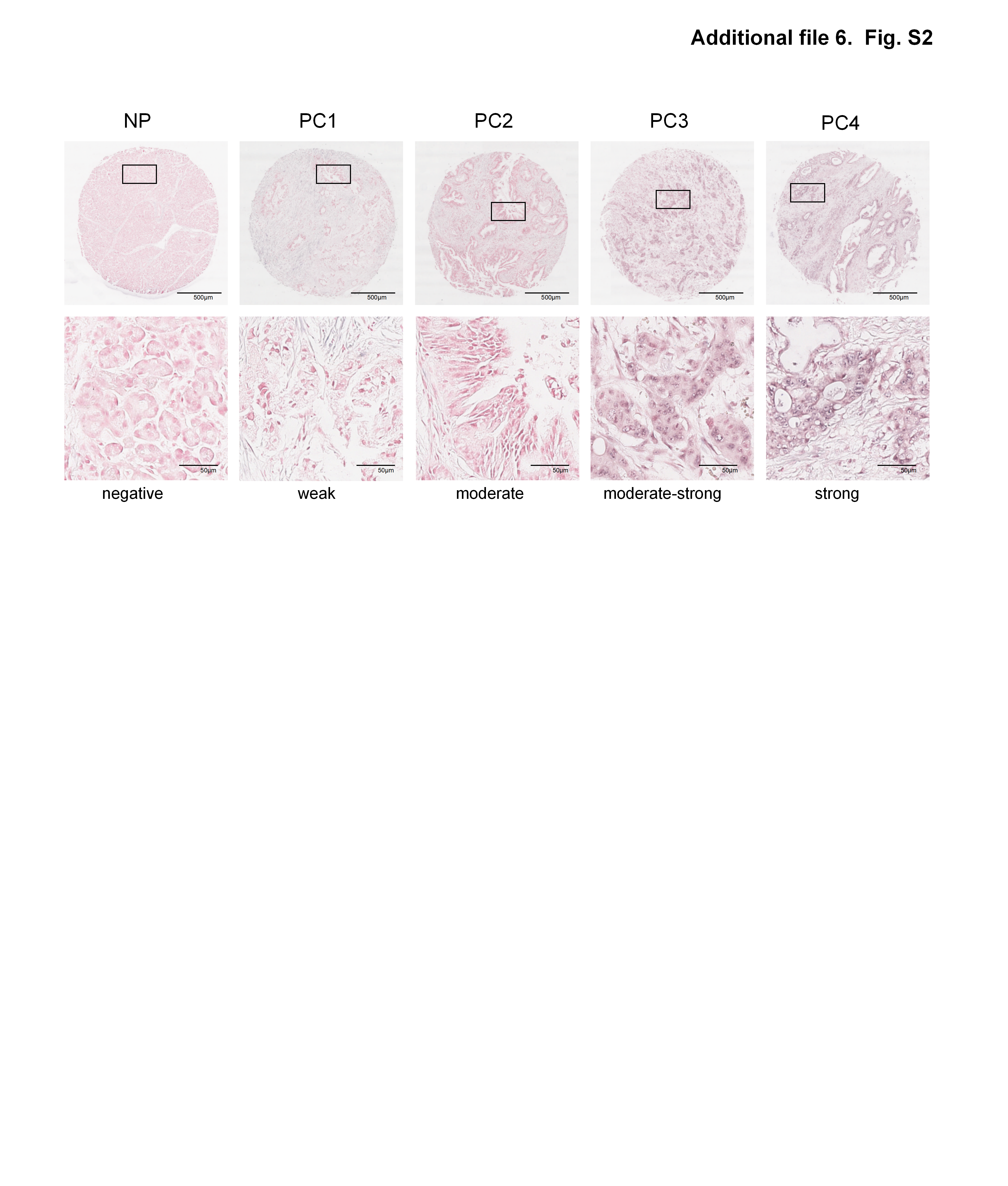

Supplement: Supplementary file 7 — Additional file 6. Fig. S2 [file 41419_2020_2617_MOESM7_ESM.tif]

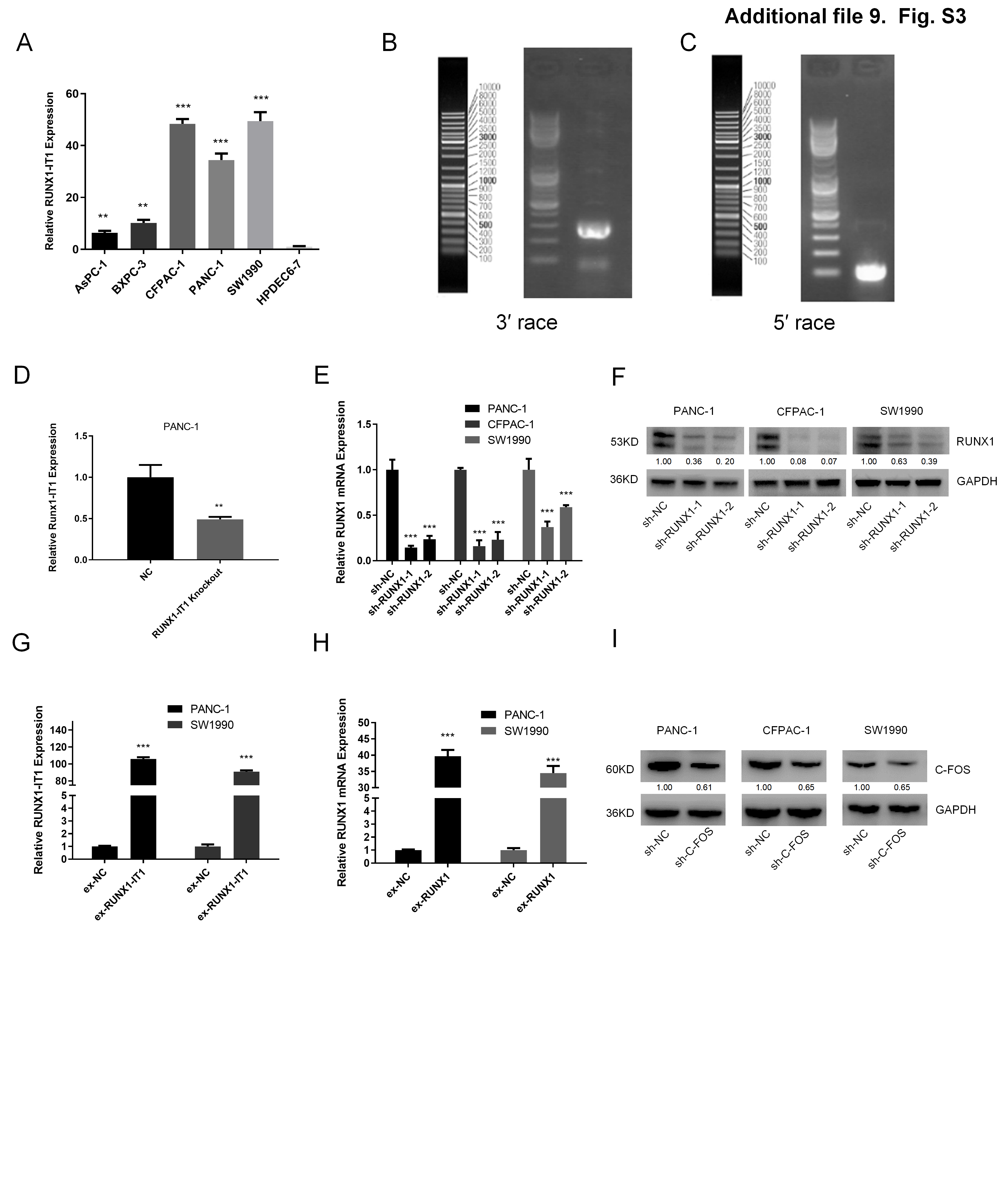

Supplement: Supplementary file 10 — Additional file 9. Fig. S3 [file 41419_2020_2617_MOESM10_ESM.tif]

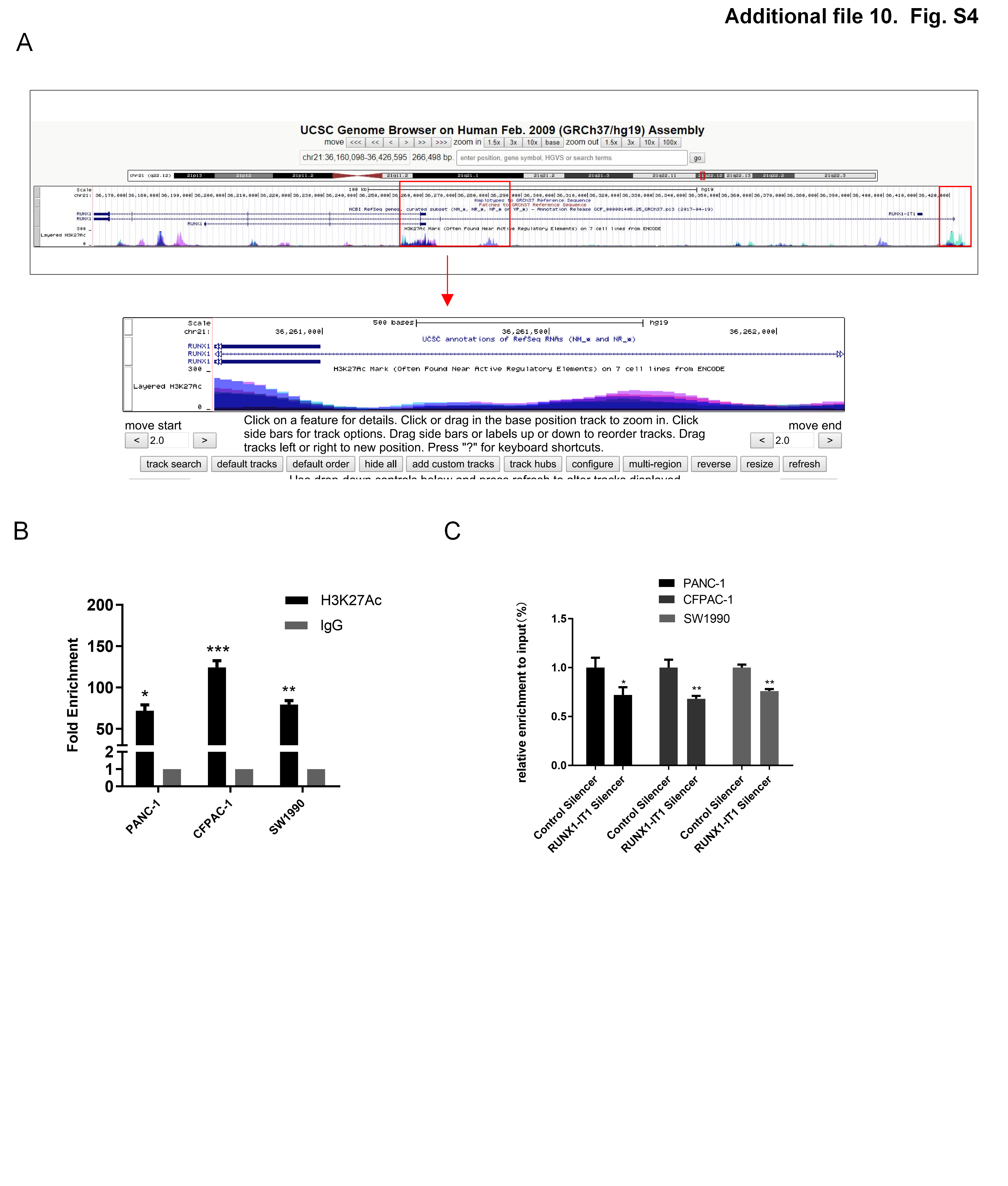

Supplement: Supplementary file 11 — Additional file 10. Fig. S4 [file 41419_2020_2617_MOESM11_ESM.tif]

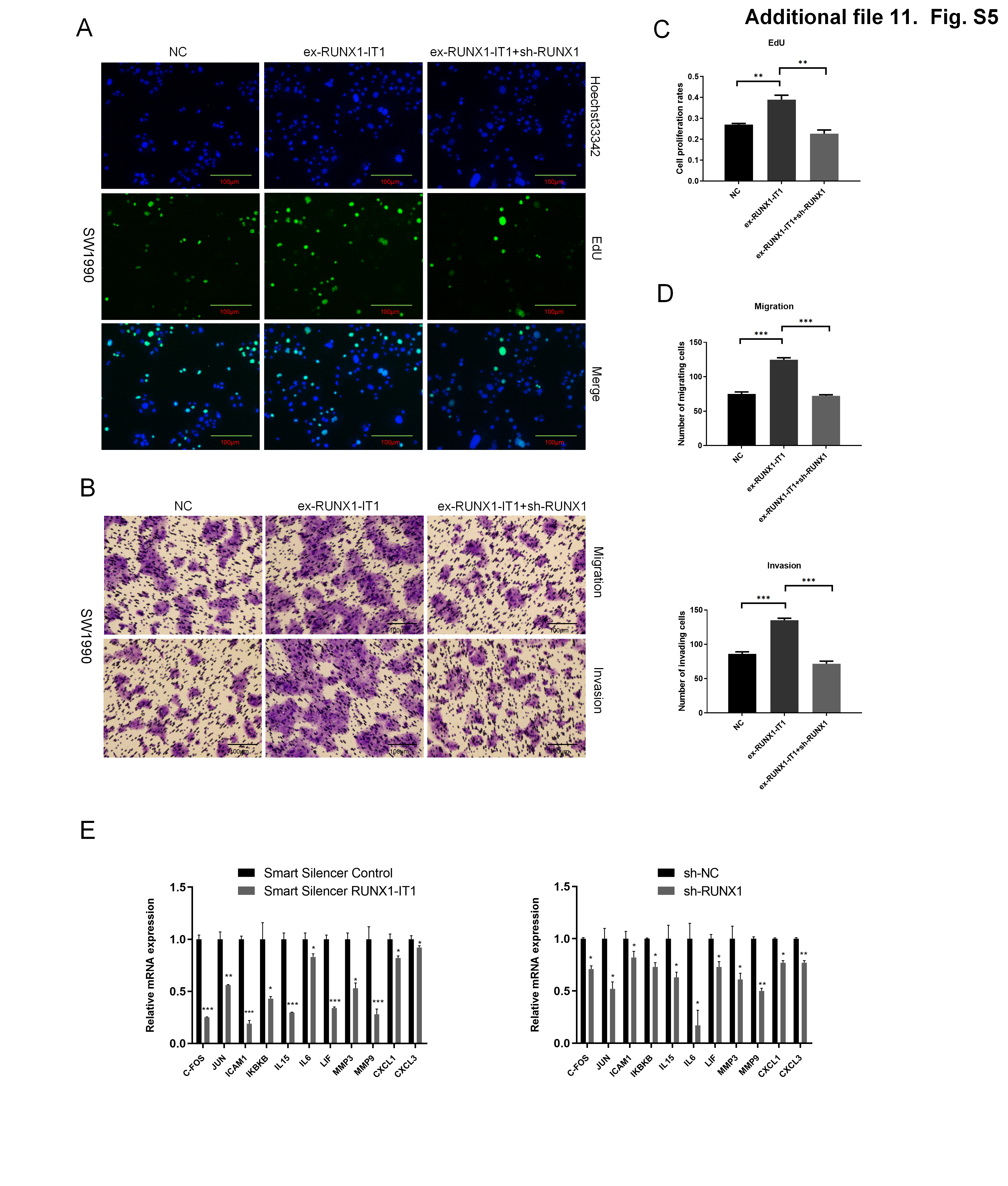

Supplement: Supplementary file 12 — Additional file 11. Fig. S5 [file 41419_2020_2617_MOESM12_ESM.tif]

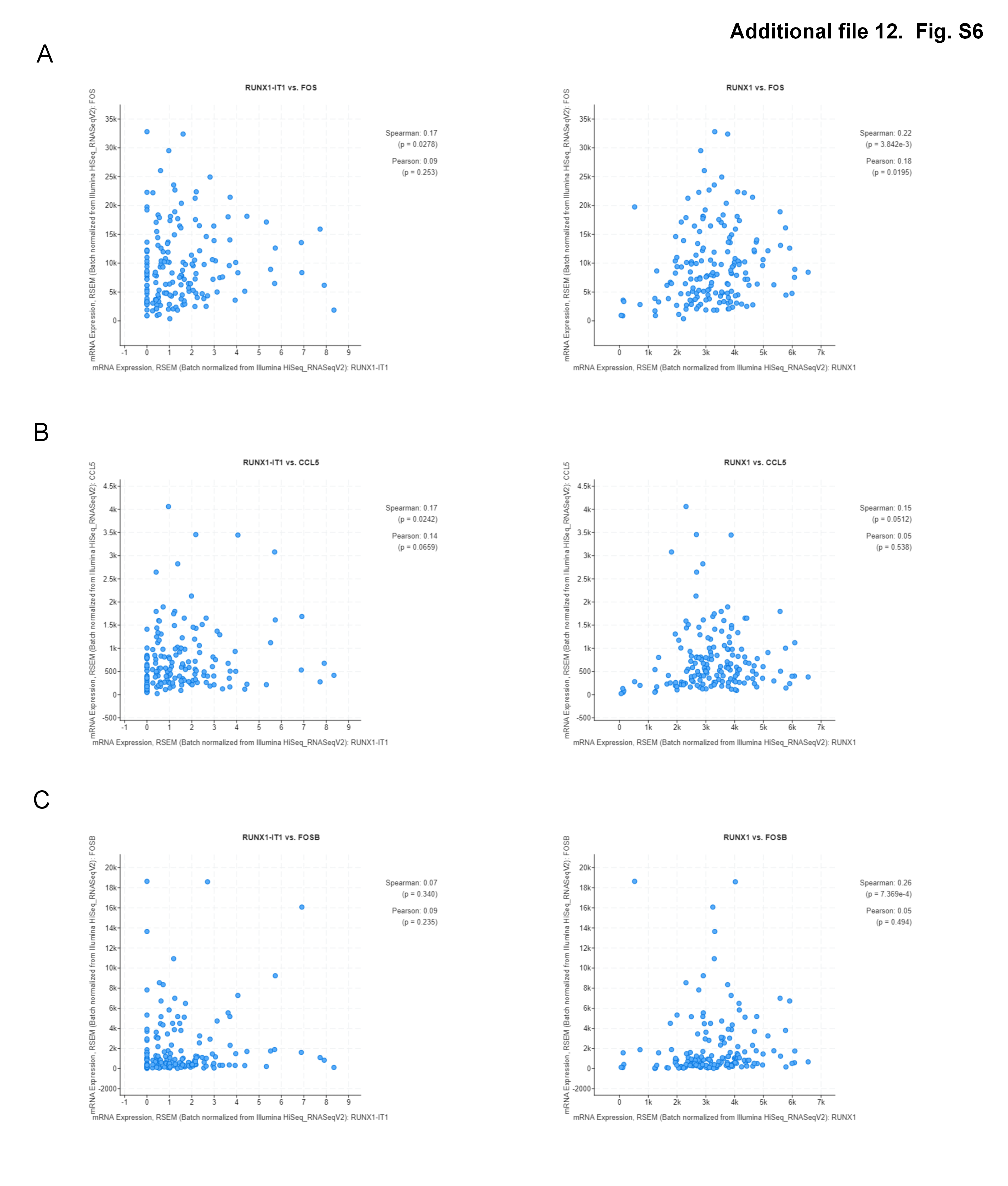

Supplement: Supplementary file 13 — Additional file 12. Fig. S6 [file 41419_2020_2617_MOESM13_ESM.tif]

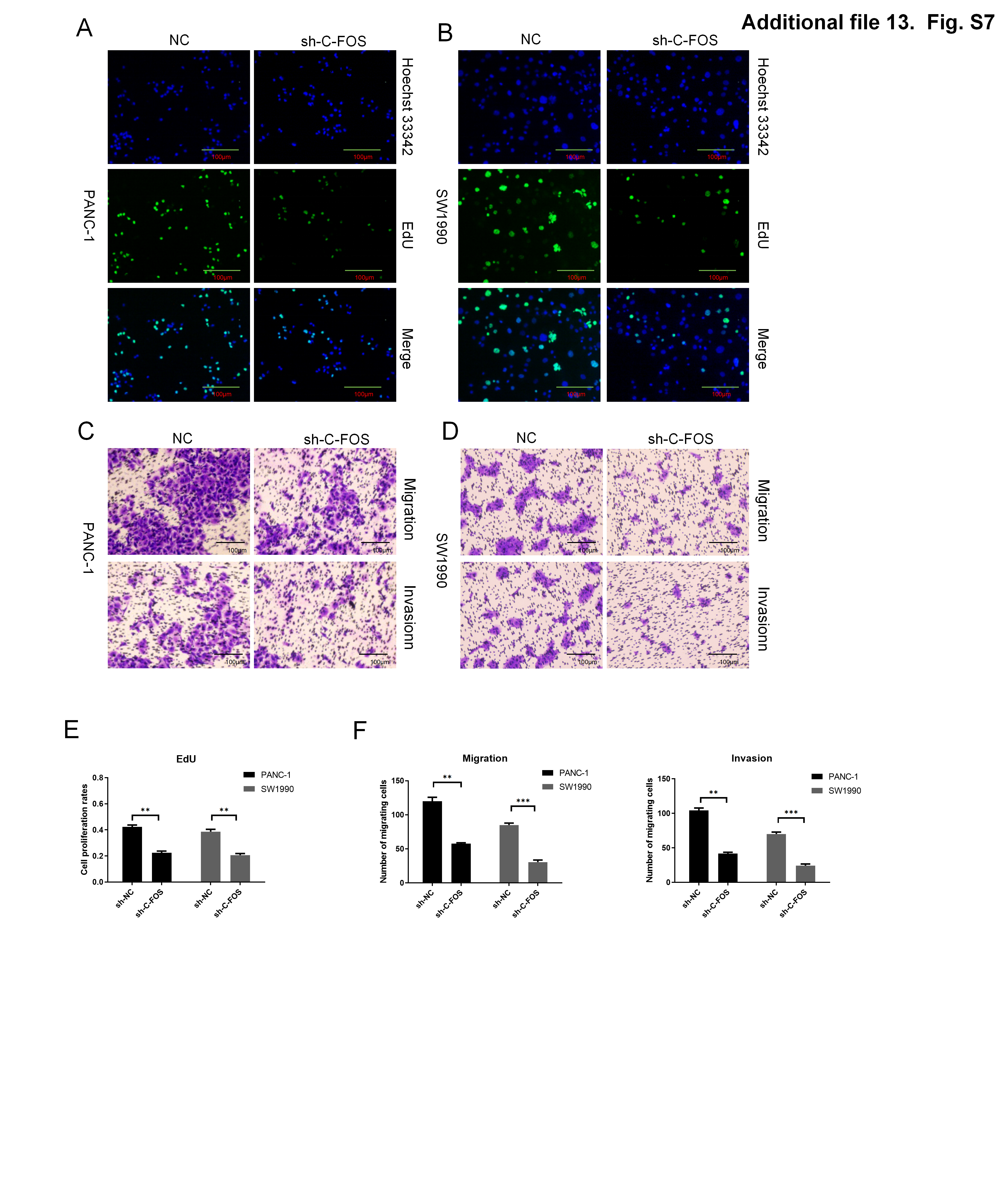

Supplement: Supplementary file 14 — Additional file 13. Fig. S7 [file 41419_2020_2617_MOESM14_ESM.tif]

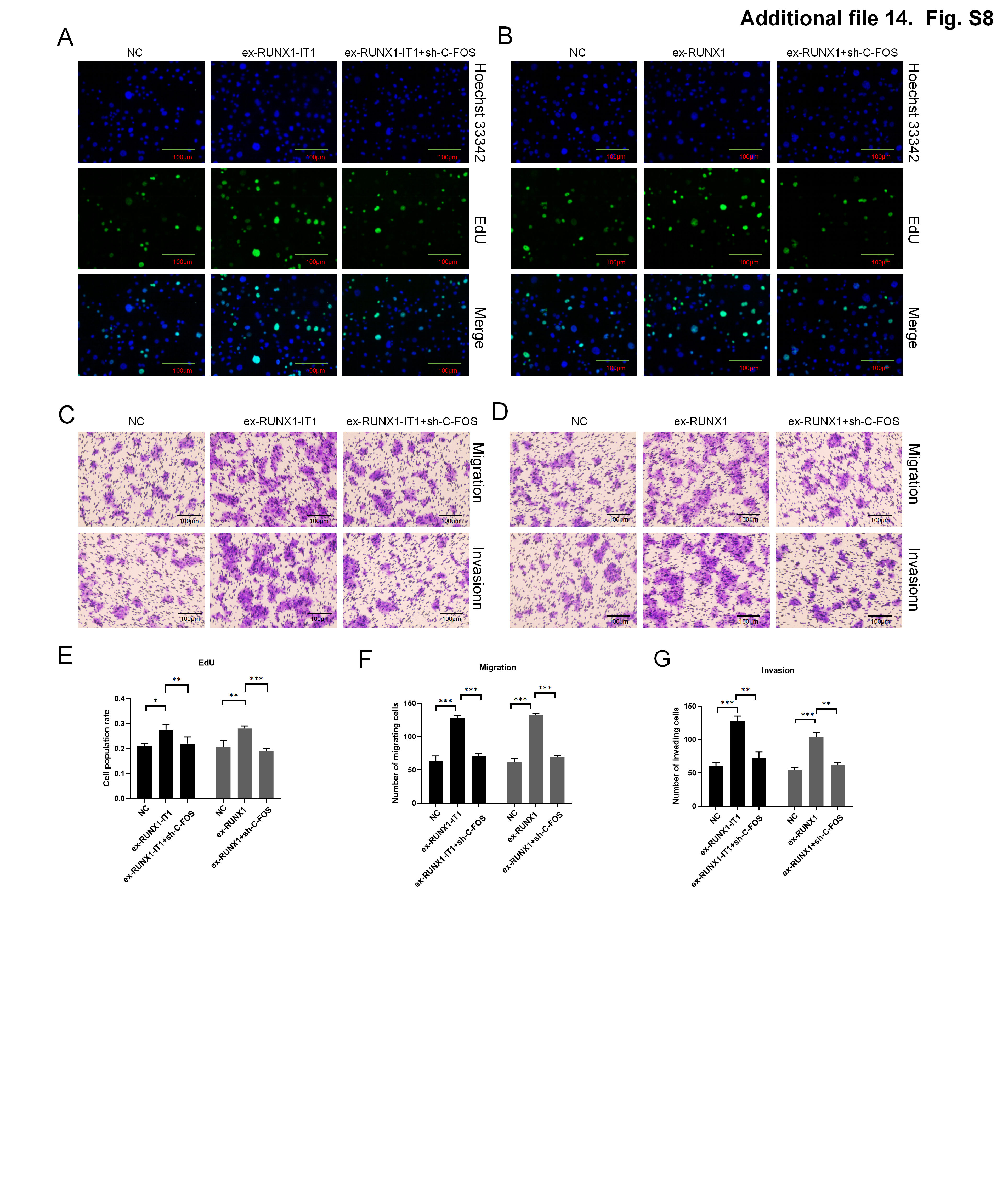

Supplement: Supplementary file 15 — Additional file 14. Fig. S8 [file 41419_2020_2617_MOESM15_ESM.tif]

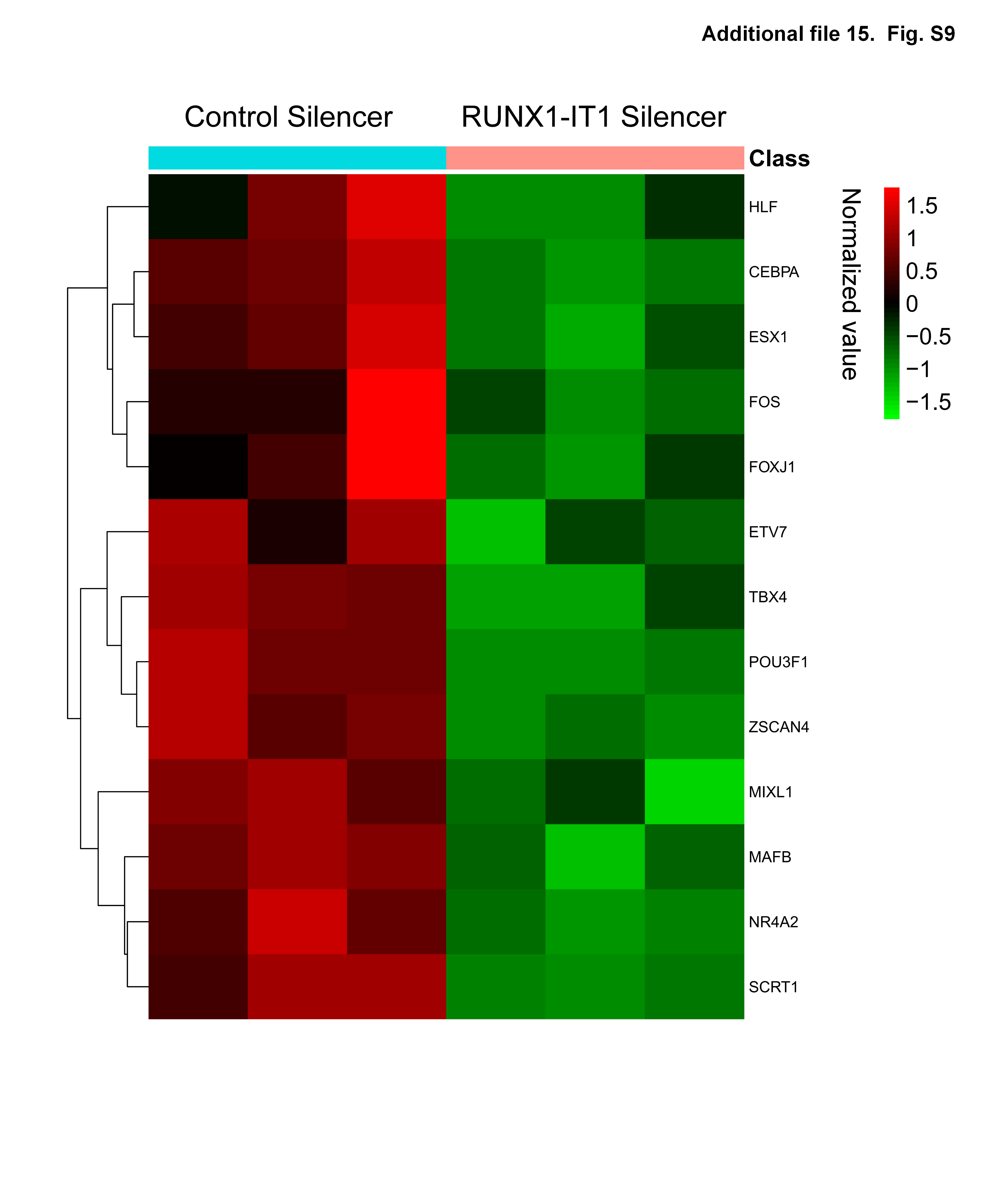

Supplement: Supplementary file 16 — Additional file 15. Fig. S9 [file 41419_2020_2617_MOESM16_ESM.tif]

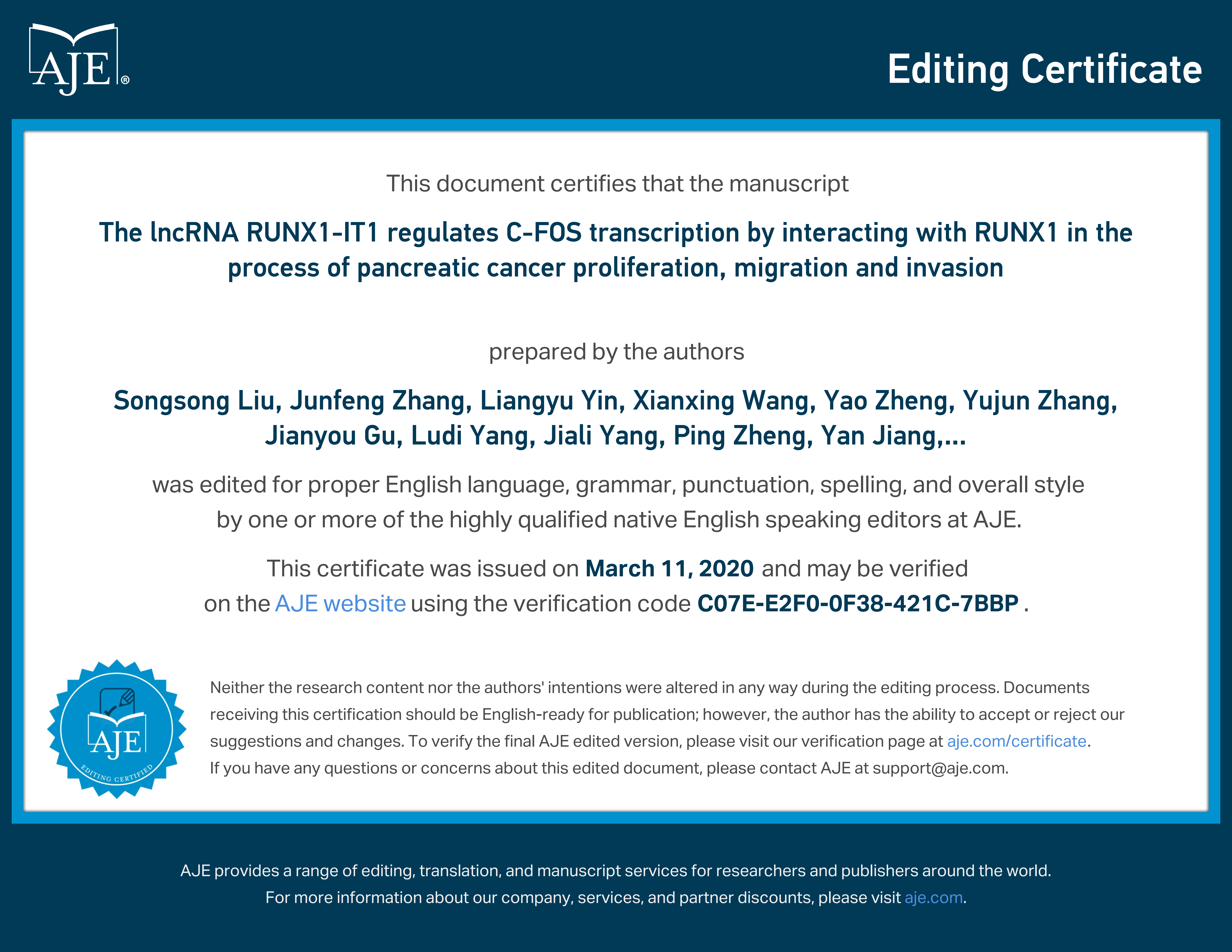

Supplement: Supplementary file 17 — Additional file 16. Fig. S10 [file 41419_2020_2617_MOESM17_ESM.tif]
