# Supplementary material for: The lncRNA RUNX1-IT1 regulates C-FOS transcription by interacting with RUNX1 in the process of pancreatic cancer proliferation, migration and invasion
Source: Cell Death Dis. 2020 Jun 2;11(6):412. doi: 10.1038/s41419-020-2617-7 (PMC7265432; doi:10.1038/s41419-020-2617-7)
Supplement: Supplementary file 6 — Additional file 5. Table S4 [file 41419_2020_2617_MOESM6_ESM.docx]

| **40 up-regulated genes and 23 down-regulated genes in three GEO datasets** | | | |
| --- | --- | --- | --- |
| **40 up-regulated genes** | **Types** | **23 down-regulated genes** | **Types** |
| **RUNX1-IT1** | **non-coding** | CELP | Pseudogene |
| EMB | coding | TDH | Pseudogene |
| MYL9 | coding | FLJ38379 | non-coding |
| CLIC6 | coding | LINC00339 | non-coding |
| CD58 | coding | DPP10-AS1 | non-coding |
| TWIST1 | coding | C8orf12 | non-coding |
| ETV1 | coding | LOC100289094 | non-coding |
| APOC1 | coding | CLPS | coding |
| PKM | coding | CHRM3 | coding |
| SKIL | coding | IL22RA1 | coding |
| RASA2 | coding | ALDH1L2 | coding |
| TNIK | coding | KIAA1324 | coding |
| EXPH5 | coding | A1CF | coding |
| CYP3A5 | coding | F11 | coding |
| ICAM1 | coding | BACE1 | coding |
| HELLS | coding | ACSM3 | coding |
| PMEPA1 | coding | ESRRG | coding |
| FXYD5 | coding | NR5A2 | coding |
| TNFAIP6 | coding | NUCB2 | coding |
| EDNRA | coding | PDE6G | coding |
| C1orf116 | coding | ART3 | coding |
| FGD6 | coding | ARHGDIG | coding |
| MX1 | coding | F2 | coding |
| CKS2 | coding |  |  |
| MBOAT2 | coding |  |  |
| BCL2A1 | coding |  |  |
| ADAM9 | coding |  |  |
| MUC5B | coding |  |  |
| SLCO1B3 | coding |  |  |
| ANO1 | coding |  |  |
| MMP1 | coding |  |  |
| IFI27 | coding |  |  |
| HK2 | coding |  |  |
| TOP2A | coding |  |  |
| NOX4 | coding |  |  |
| ASPM | coding |  |  |
| C19orf33 | coding |  |  |
| GPRC5A | coding |  |  |
| LEMD1 | coding |  |  |
| MSLN | coding |  |  |
