# Supplementary material for: The lncRNA RUNX1-IT1 regulates C-FOS transcription by interacting with RUNX1 in the process of pancreatic cancer proliferation, migration and invasion
Source: Cell Death Dis. 2020 Jun 2;11(6):412. doi: 10.1038/s41419-020-2617-7 (PMC7265432; doi:10.1038/s41419-020-2617-7)
Supplement: Supplementary file 8 — Additional file 7. Table S5 [file 41419_2020_2617_MOESM8_ESM.docx]

**Table 1 Statistical analysis of the correlation between RUNX1-IT1 expression and clinical characteristics of PC.**

| Characteristics | Pancreatic adenocarcinoma cases (N=175) | | |
| --- | --- | --- | --- |
|  | IT1 high (n=118) | IT1 low (n=57) | *P* |
| Age | 59.56 (10.67) | 58.70 (9.54) | 0.607 |
| Gender, n (%) |  |  | 0.655 |
| Male | 74 (62.7) | 33 (57.9) |  |
| Female | 44 (37.3) | 24 (42.1) |  |
| Tumor size, M (IQR) | 8.40 (5.03, 21.00) | 8.30 (4.00, 17.50) | 0.425 |
| Differentiation |  |  | 0.005* |
| High | 12 (10.2) | 12 (21.1) |  |
| Medium | 66 (55.9) | 38 (66.7) |  |
| Poor | 40 (33.9) | 7 (12.2) |  |
| Clinical stage, AJCC 8^th^ |  |  | 0.007* |
| I-II | 72 (61.0) | 47 (82.5) |  |
| III-IV | 46 (39.0) | 10 (17.5) |  |
| Peri-nerual invasion |  |  | 0.432 |
| No | 85 (72.0) | 37 (64.9) |  |
| Yes | 33 (28.0) | 20 (35.1) |  |
| Lymphnode invasion |  |  | 0.001* |
| No | 74 (62.7) | 50 (87.7) |  |
| Yes | 44 (37.3) | 7 (12.3) |  |
| Local metastasis |  |  | 0.972 |
| No | 108 (91.5) | 53 (93.0) |  |
| Yes | 10 ( 8.5) | 4 (7.0) |  |

** P<0.05; AJCC, American Joint Committee on Cancer*
