# Supplementary material for: The lncRNA RUNX1-IT1 regulates C-FOS transcription by interacting with RUNX1 in the process of pancreatic cancer proliferation, migration and invasion
Source: Cell Death Dis. 2020 Jun 2;11(6):412. doi: 10.1038/s41419-020-2617-7 (PMC7265432; doi:10.1038/s41419-020-2617-7)
Supplement: Supplementary file 9 — Additional file 8. Table S6 [file 41419_2020_2617_MOESM9_ESM.docx]

**Table 2** **Univariate and multivariate Cox regression analyses of the association between RUNX1-IT1 expression and survival.**

| Characterastics |  | Univariate | |  | Multivariate | |
| --- | --- | --- | --- | --- | --- | --- |
|  |  | HR (95%CI) | *P* |  | HR (95%CI) | *P* |
| Age, continuous |  | 1.01 (0.99-1.03) | 0.358 |  | - | - |
| Gender (male vs. female) |  | 1.0 (0.68-1.48) | 0.994 |  | - | - |
| Differentiation |  |  |  |  |  |  |
| medium vs. high |  | 1.8 (0.90-3.62) | 0.099 |  | 2.03 (1.00-4.11) | 0.049 |
| poor vs. high |  | 2.5 (1.20-5.24) | 0.015 |  | 2.19 (1.04-4.61) | 0.039 |
| Lymphnode (n1 vs. n0) |  | 1.92 (1.26-2.93) | 0.002 |  | - | - |
| Clinical stage (III-IV vs. I-II) |  | 1.56 (1.19-2.06) | 0.002 |  | 1.56 (1.18-2.07) | 0.002 |
| RUNX1-IT1 (high vs. Low) |  | 2.98 (1.9-4.68) | <0.001 |  | 2.96 (1.87-4.69) | <0.001 |

*HR denotes hazard ratio.*
